# Beyond skin-deep: targeting the plant surface for crop improvement

**DOI:** 10.1093/jxb/erad321

**Published:** 2023-08-17

**Authors:** Jenna Bryanne Jolliffe, Stefania Pilati, Claudio Moser, Justin Graham Lashbrooke

**Affiliations:** South African Grape and Wine Research Institute, Stellenbosch University, Stellenbosch, 7600, South Africa; Research and Innovation Centre, Edmund Mach Foundation, San Michele all’Adige, 38098, Italy; Research and Innovation Centre, Edmund Mach Foundation, San Michele all’Adige, 38098, Italy; Research and Innovation Centre, Edmund Mach Foundation, San Michele all’Adige, 38098, Italy; South African Grape and Wine Research Institute, Stellenbosch University, Stellenbosch, 7600, South Africa; Department of Genetics, Stellenbosch University, Stellenbosch, 7600, South Africa; University of Sydney, Australia

**Keywords:** Biotechnology, crop improvement, cuticle, drought tolerance, epidermal layer, fruit quality, new breeding techniques (NBTs), stomata, trichome

## Abstract

The above-ground plant surface is a well-adapted tissue layer that acts as an interface between the plant and its surrounding environment. As such, its primary role is to protect against desiccation and maintain the gaseous exchange required for photosynthesis. Further, this surface layer provides a barrier against pathogens and herbivory, while attracting pollinators and agents of seed dispersal. In the context of agriculture, the plant surface is strongly linked to post-harvest crop quality and yield. The epidermal layer contains several unique cell types adapted for these functions, while the non-lignified above-ground plant organs are covered by a hydrophobic cuticular membrane. This review aims to provide an overview of the latest understanding of the molecular mechanisms underlying crop cuticle and epidermal cell formation, with focus placed on genetic elements contributing towards quality, yield, drought tolerance, herbivory defence, pathogen resistance, pollinator attraction, and sterility, while highlighting the inter-relatedness of plant surface development and traits. Potential crop improvement strategies utilizing this knowledge are outlined in the context of the recent development of new breeding techniques.

## Teaser

This review describes our current knowledge of the genes associated with plant cuticle and epidermal layer formation in crop plants, and how to harness this knowledge for crop improvement.

## Introduction

The epidermis of above-ground plant organs is derived from the L1 tissue present at the shoot apical meristem (Soyars *et al*., 2016). This multifunctional layer differentiates to form specialized cell types, such as stomatal guard cells and trichomes, as well as pavement cells which, despite being less specialized, are important for ensuring the correct patterning and functionality of the former (Glover, 2000; Javelle *et al.*, 2011). In turn, the plant epidermal cells synthesize and assemble a protective external waxy layer known as the cuticle (Schreiber, 2010), which together contribute towards many significant crop traits.

Stomatal guard cells regulate the plant–environment gaseous exchange by altering turgidity. This function is not only important for water conservation, but is also required for leaf cooling, CO_2_ uptake, and the transport of solutes within the transpiration stream. Under short-term water deficit conditions, loss of guard cell turgor, induced by abscisic acid (ABA) signalling (Sussmilch *et al.*, 2019), leads to stomatal closure and reduced transpirational water loss. Extended exposure to drought stimuli, however, promotes decreased stomatal density in plants which can lead to an improved water-use efficiency (WUE) (Caine *et al.*, 2019). The regulatory pathways that drive stomatal formation are well characterized in Arabidopsis and have been shown to differ significantly between monocots and dicots (Buckley *et al.*, 2020).

Hair-like protuberances, known as trichomes, are commonly found on the surface of plant organs. These surface features are classified based on their morphology and secretory ability as either unicellular or multicellular, glandular or non-glandular, as well as branched or unbranched, which are traits driven by diverse regulatory programmes (Liu *et al.*, 2016; Z. Wang *et al.*, 2019; Chalvin *et al.*, 2020). Trichomes perform a diverse range of important plant functions. Glandular trichomes, for example, are able to synthesize and secrete commercially significant metabolites such as artemisinin, essential oils, and cannabinoids, to name a few, making them useful targets for metabolic engineering or biopharming (Schuurink and Tissier, 2020). The focus of the current review, however, will be aimed towards trichome functions related to crop protection and market value.

The cuticle layer acts primarily as a hydrophobic barrier against plant dehydration, though also contributing towards post-harvest crop quality, disease resistance, and pollen development (Lara *et al.*, 2019; Wan *et al.*, 2020; Arya *et al.*, 2021; L. Liu *et al.*, 2022; Wang and Chang, 2022). The cuticle composition and structure vary amongst plant species, cultivars, organs, and developmental stages, but it is essentially composed of the cutin polymer, cuticular waxes, and, in many cases, secondary metabolites (Martin and Rose, 2014). Pathways driving the biosynthesis and transportation of these essential cuticular components are tightly regulated by an intricate network involving transcription factors (TFs), signalling proteins, and post-transcriptional modulation (Borisjuk *et al.*, 2014; Hen-Avivi *et al.*, 2014). Suberin is another closely related polymer which shares common biosynthetic precursors with cutin and wax (Philippe *et al.*, 2020). Importantly, it has been shown that while the cuticular layer functions primarily as a physical barrier it also mediates osmotic stress tolerance through the regulation of ABA biosynthesis and signalling (Z.Y. Wang *et al.*, 2011).

Knowledge of the molecular and cellular biology of above-ground surface formation allows one to directly or indirectly infer key genes that can be targeted for modulation via biotechnological means, enabling the production of crop lines better adapted to agricultural needs. The advent of new breeding techniques has made genetic modulation of surface-related genes significantly more attainable in crop species. However, careful consideration must be given when deciding on the type of gene modification (e.g. loss-of-function) or biotechnology method (e.g. transgenesis) used to achieve the desired trait. Regardless of the technique implemented, all approaches may be limited by the regenerative capacity of the crop of interest. In addition, it is important to be thorough when selecting candidate genes for modification so as to avoid unwanted pleiotropic or off-target traits (Basso *et al.*, 2019). This phenomenon is particularly relevant in plant surface manipulation since genes involved in surface formation tend to display overlapping and interconnected functions (Torii, 2021).

**Fig. 1.**
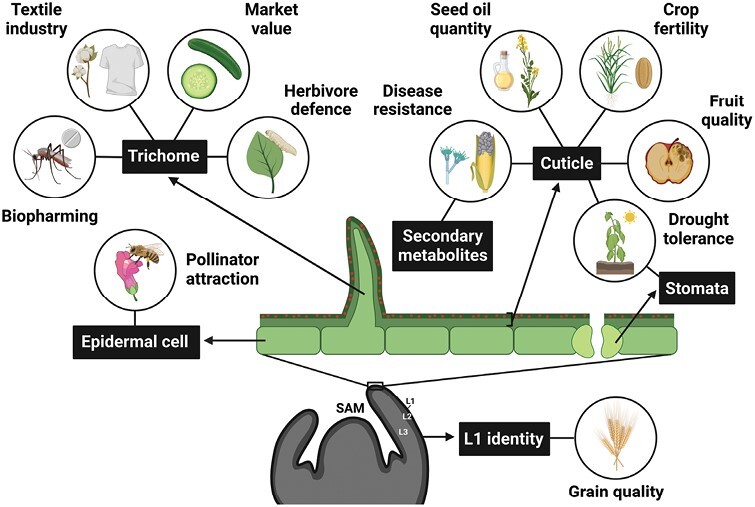
Simplified schematic cross-section of above-ground plant surface elements and the crop traits to which they contribute. Surface elements are highlighted by black boxes and include the L1 layer/identity, epidermal cells, trichomes, stomata, as well as the cuticle layer which is made up of three components: the cutin polymer (light green layer), cuticular waxes (dark green layer), and secondary metabolites (red dots). SAM, shoot apical meristem.

This review provides a summary of the current understanding regarding the genetic and molecular basis of above-ground plant surface formation and the important role it plays in crop production, with significant emphasis placed on target genes that may be manipulated via biotechnological approaches to drive crop improvement. Crop genes are described in the context of a desired phenotypic alteration, with specific consideration given to quality, yield, drought tolerance, biotic stress tolerance, pollinator attraction, and fertility (Fig. 1).

## Crop quality and yield

### Surface traits impacting cereal production

**Fig. 2.**
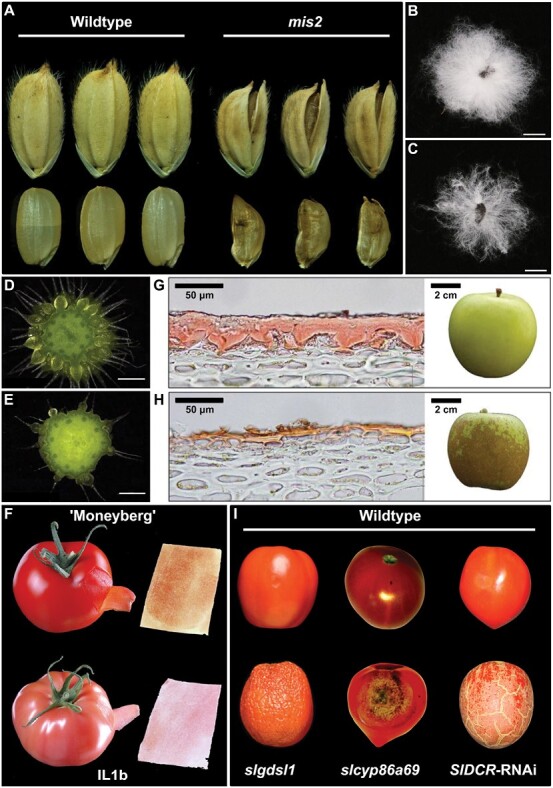
Contribution of surface-related genes to different crop quality traits. (A) The L1-related *mis2* mutant developed rice grains that were irregularly shaped with an open-hull phenotype (Chun *et al.*, 2020). *GhMML4*-silenced (C) cotton seeds showed reduced fibre densities compared with the wild type (B) (Wu *et al.*, 2018). Decreased fruit spine density in *CsMYB6*-OE (E) cucumber compared with the wild type (D) (L. Zhao *et al.*, 2020). (F) The pink-coloured fruit phenotype of IL1b resulting from the down-regulation of *SlMYB12* and, subsequently, a lack of naringenin chalcone in the peel (Ballester *et al.*, 2010). *MdSHN3* is associated with apple russet formation with low and high expression levels driving regular (G) and impaired (H) cuticle accumulation, respectively (Lashbrooke *et al*., 2015b). (I) Enzymes involved in cuticle biosynthesis which have been linked to fruit dehydration (*slgdsl1*; Isaacson *et al.*, 2009), fruit decay (*slcyp86a69*; Shi *et al.*, 2013), as well as microcracks and fissures (*SlDCR*-RNAi; Lashbrooke *et al.*, 2016) in tomato.

The quality and yield of grains are affected by the functionality of L1-related *CRINKLY4* (*CR4*) and *DEFECTIVE KERNEL1* (*DEK1*) orthologous genes (Supplementary Table S1). In rice (*Oryza sativa*), *OsCR4* knockdown lines developed abnormal cells with a discontinuous cuticle in the inner and outer epidermal layers. This phenotype led to distortions in the protective interlocking barrier between the palea and lemma, and, as a result, disruptions in pistil pollination (Pu *et al.*, 2012). In addition, *cr4* knockout mutants developed smaller rice grains with undesirable wrinkled surfaces (Fig. 2A; Chun *et al.*, 2020). Mutations in the DEK1-related ADAXIALIZED LEAF1 (ADL1) protein have been linked to defective epidermal phenotypes related to bulliform cell patterning and, consequently, irregular leaf rolling patterns which can lead to grain yield reductions (Hibara *et al.*, 2009; Zou *et al.*, 2011). Bulliform cells are large vacuolated epidermal cells that feature on the adaxial leaf surfaces of monocot crops (Kadioglu *et al.*, 2012). During water scarcity, loss in bulliform cell turgidity promotes adaxial leaf rolling which reduces sunlight exposure, and thus water loss. Comparable leaf curling phenotypes have been observed for the L1-associated *CURLY FLAG LEAF1* (*CFL1*) gene which negatively regulates cuticle development in rice (Wu *et al.*, 2011). Interestingly, despite no changes reported for the leaf curling patterns of *cr4* rice mutants, a significant down-regulation of the leaf rolling-associated *ROC5* gene was found (Zou *et al.*, 2011; Chun *et al.*, 2020). This ROC5 HD-ZIP IV protein has been shown to affect leaf curling characteristics through the direct regulation of cuticle genes, such as *OsCYP96B4* (X. Zhang *et al.*, 2021). Since similar epidermal phenotypes have been reported for the *CR4* and *DEK1* maize (*Zea mays*) genes (Becraft *et al.*, 1996, 2002; Jin *et al.*, 2000), it is expected that these genes also play crucial roles in maize quality and yield.

Naked or ‘hull-less’ barley (*Hordeum vulgare*) is preferred to hulled barley since the loosely covering husks of the caryopses are more readily separated upon threshing. In addition, it has been found that naked barley is richer in nutritional constituents, such as β-glucan and proteins, and contains lower insoluble dietary fibre (Boros *et al.*, 1996). In barley, the naked phenotype has been linked to the function of the cuticle-related *SHN1* gene orthologue, *Nud*. It is suggested that the Nud TF regulates lipid deposition between the caryopsis and inner side of the hull to drive organ adhesion (Taketa *et al.*, 2008).

The formation of trichomes (macro- and microhairs) on the leaf and glume surfaces of rice can lead to difficulties related to allergies and itchiness during manual harvests, as well as limit the packing capacity of the crop (Li *et al.*, 2012). As such, great emphasis has been placed on breeding rice varieties with glabrous traits. Multiple studies have shown that loss-of-function mutations in the *WUSCHEL-like* homeobox *OsWOX3B* gene are associated with smoother hull surfaces that are deficient in trichomes (Zhang *et al.*, 2012). In addition, Sun *et al.* (2017) found that OsWOX3B directly binds to the promoter region of the trichome elongation-related *HAIRY LEAF6* (*HL6*) gene, while an allelic *HL6* variant (*OsGLABRA6*) has been associated with trichome initiation (Y. Xie *et al.*, 2020).

### Rapeseed oil quantity

Crops such as rapeseed (*Brassica napus*) are important resources for vegetable oil production and high-quality animal feed proteins, while also providing raw materials for industrial products such as biodiesel (Zhai *et al.*, 2020). For this reason, studies have focused on understanding the factors that can affect the quality and quantity of rapeseed oil. One surface-related example is the *BnWIN1* gene, which has been shown to play a dual role in intracellular and extracellular lipid synthesis (Liu *et al.*, 2019). In *BnWIN1*-overexpressing (OE) lines, leaf wax load is increased under salt stress without impacting seed oil content. In addition, seed oil content is increased under normal growth conditions. BnWIN1 acts by modulating the expression of both wax and oil biosynthetic genes (Liu *et al.*, 2019).

### Fibre quality of cotton seeds for textile purposes

Cotton (*Gossypium* spp.) forms seed trichomes (fuzz or lint fibres) that are an essential resource for the textile industry. The value of cotton fibres is based on traits such as yield and length, which are dependent on the initiation and elongation phases of fibre development, respectively (Deng *et al.*, 2012). Cotton fibres are morphologically comparable with Arabidopsis trichomes since they are unicellular. For this reason, it is unsurprising that cotton fibre initiation is also driven by a MYB–bHLH–WD40 (MBW) activation complex. In support of this, the cotton *GLABRA1* (*GL1*) (*GaMYB2* and *GhMYB3*), *TRANSPARENT TESTA GLABRA1* (*GhTTG1* and *GhTTG3*), and *GL3*/*ENHANCER OF GL3* (*GhDEL65*) gene homologues were shown to fully, or at least partially, rescue their respective Arabidopsis mutants (Wang *et al*., 2004; Humphries *et al.*, 2005; Shangguan *et al.*, 2016, 2021). In addition, the GhDEL65 protein directly interacted with both GhMYB2 and GhTTG3 (Shangguan *et al.*, 2016). Acting downstream of the MBW complex, a *GL2* homologue (*GaHOX1*) was found to rescue the trichome phenotype of *atgl2-2* mutants, while the *GL2*-related *GhHOX3* gene positively regulated fibre elongation, leading to longer lint fibres in overexpressing lines (Guan *et al.*, 2008; Shan *et al.*, 2014). This phenotype is not only important for cotton yield, but also improves the fibre quality since shorter fuzz fibres do not yield spinnable thread. The cotton CAPRICE (GhCPC) TF negatively regulates the MBW complex by interacting with GhTTG1 and GhMYC1, with the latter protein directly binding to the promoter region of the *GhHOX3* gene (Liu *et al*., 2015). L1-specific genes also play a critical role in driving the specification of fibre cells (Supplementary Table S1). This was observed for homologues of the *ARABIDOPSIS THALIANA MERISTEM L1* (*ATML1*) gene (*GhHD*-*1* and *GbML1*) which increased the number of fibre initials in overexpressing cotton and Arabidopsis lines, respectively (Zhang *et al.*, 2010; Walford *et al.*, 2012). In the case of *GhHD*-*1*, this also led to an undesirable increase in fuzz fibre percentage. The cotton *PROTODERMAL FACTOR1* (*GbPDF1*) gene reduced fibre initiation and resulted in shorter fibres with a lower percentage of lint in knockdown lines (Deng *et al*., 2012). The *MIXTA*-related *GhMYB25* and *GhMYB25-like* genes were associated with shorter fibre and nearly fibreless seed phenotypes, respectively, in silenced cotton (Machado *et al.*, 2009; Walford *et al.*, 2011). Consistent with these phenotypes, GhMYB25-like was found to act upstream of GhMYB25 which, in turn, positively regulated the GL1-related GhMYB109 protein involved in fibre length control (Pu *et al.*, 2008; Machado *et al.*, 2009; Walford *et al.*, 2011). It is important to note that MIXTA-related proteins promote different fibre cell types. For instance, GhMYB25-like drives fuzz fibre initiation (Wan *et al.*, 2016), while GhMML4 is linked to higher quality lint fibre (Fig. 2B, C; Wu *et al.*, 2018). Cotton fibre formation is modulated by a variety of phytohormones, which has recently been reviewed by Tian and Zhang (2021).

### Market-driven cucumber fruit spines

The size and density of fruit spines (trichomes) are important traits determining the target market and value of cucumber fruit (*Cucumis sativus*). Indeed, cucumbers with a higher density of small fruit spines are popular amongst consumers in China, while almost entirely glabrous fruit surfaces are preferred by the European market. In addition, large and sparse fruit spines are targeted for American pickle varieties (Pan *et al.*, 2015). In terms of market value, some cucumbers develop fruit spines in combination with tubercules, which result in a warty phenotype that is considered less desirable than its non-warty counterpart due to taste and storability (Yang *et al.*, 2014). The multicellular fruit trichomes of cucumber are formed, in part, through the function of the cucumber *GLABROUS1* (*CsGL1*) gene, encoding an HD-ZIP I family member (Li *et al.*, 2015). Cucumber *gl1* mutants display apparently glabrous phenotypes, although papillae are still present on the fruit surfaces (Li *et al.*, 2015). This phenotype indicates an important role for *CsGL1* in trichome differentiation. In addition, studies have shown that *CsGL1* is epistatic over the C2H2 zinc finger gene *Tu*/*DULL*, which contributes to the lack of tubercules present on *csgl1* mutant fruits (Yang *et al.*, 2014). The L1-related cucumber *GLABROUS3* (*CsGL3*) gene, encoding an HD-ZIP IV protein, was identified as a positive regulator of trichome cell specification since loss-of-function mutants developed plant surfaces that were devoid of trichomes (Pan *et al.*, 2015). Chen *et al.* (2016) showed that the WD-repeat protein CsTTG1 promotes trichome initiation, with silenced cucumber lines developing fewer fruit trichomes. Further, CsTTG1 interacts with CsGL1 to drive tubercule formation, thus having an additional function in warty fruit phenotypes (Chen *et al.*, 2016). With regards to negative regulators, the MIXTA-like protein CsMYB6 was found to reduce spine and tubercule initiation, in overexpressing cucumber fruit, by interacting with CsTTG1 and CsTu/DULL, respectively (Fig. 2D, E, L; L; Zhao *et al*., 2020). In addition, the cucumber TRIPTYCHON (CsTRY) TF is predicted to negatively regulate trichome initiation by forming a protein complex with CsMYB6 (Yang *et al.*, 2018). For a detailed discussion on cucumber spine types, stages of spine formation, and hormone-related regulatory interactions, we refer the reader to the reviews of Liu *et al.* (2016) and Han *et al.* (2022).

### Cuticle-related fruit colour development

Pink tomato fruit (*Solanum lycopersicum*) are preferred by some consumers, particularly in Asian markets, due to the notion that they possess a greater palatability (Zhu *et al.*, 2018). In tomato, the major flavonoid, naringenin chalcone, which accumulates exclusively in the fruit cuticle during ripening, contributes to the typical orange-red pigment of mature fruit. Silencing the tomato CHALCONE SYNTHASE (SlCHS) enzymes, which drive the synthesis of naringenin chalcone, leads to the development of pink-coloured fruit with colourless cuticles that possess a reduced viscoelastic behaviour and thickness (España *et al.*, 2014). Acting as a key regulator of this flavonoid pathway is the R2R3-MYB protein SlMYB12 which was shown to underlie the peel-specific *yellow* (*y*) mutant phenotype in tomato. In *y* mutants, a significant reduction in naringenin chalcone content was strongly correlated with the expression level of the *SlMYB12* gene and several flavonoid-related genes, including *SlCHS* (Fig. 2F) (Adato *et al.*, 2009; Ballester *et al.*, 2010).

These, however, are not the only ripening-related genes that are known to modify fruit cuticle properties. Additional examples include: the *NON-RIPENING* (*NOR*) and *RIPENING INHIBITOR* (*RIN*) genes which alter the cuticular wax profiles of tomato mutants during fruit development (Kosma *et al.*, 2010); the MADS-box TOMATO AGAMOUS-LIKE1 (TAGL1) TF which promotes thicker, albeit weaker, tomato fruit cuticles in overexpressing lines by regulating cutin biosynthesis (Giménez *et al.*, 2015); as well as the two tomato *FRUITFULL* (*FUL*) gene orthologues (*SlFUL1* and *SlFUL2*) which form orange-ripe fruit phenotypes, due to reduced cuticle lycopene, along with the down-regulation of cuticle-related genes in double knockdown lines (Bemer *et al.*, 2012). Together, these studies illustrate the interdependent regulation between fruit ripening, cuticle formation, and post-harvest quality traits.

### Cuticle failure, suberin, and fruit russeting

The cuticle plays an important role in providing external mechanical support to rapidly expanding fruit tissues during development. However, when the extendibility of the cuticle is exceeded, due to environmental stresses or cutin deficiency, mechanical failure of the fruit surface can occur, resulting in the development of microcracks (cuticular) or macrocracks (subcuticular). These surface wounds induce suberin deposition which leads to skin disorders such as fruit russeting. While suberized fruit surfaces are often considered a disorder, russeted or reticulated surfaces are commonly found in varieties of apple (*Malus*×*domestica*), cucumber, melon (*Cucumis melo*), and pear (*Pyrus pyrifolia*) (Lashbrooke *et al.*, 2015b; Cohen *et al.*, 2019; Arya *et al.*, 2022; Zhang *et al.*, 2023). Studies of such varieties have led to the identification of genes involved in or regulating the cutin and suberin biosynthetic pathways. Indeed, orthologues of the MYB107 and MYB9 TFs have been found to positively regulate suberin deposition in several crop species and organs (Lashbrooke *et al*., 2016).

In melon, skin reticulation is considered a positive fruit trait since the netting phenotype is widely accepted by consumers and can increase the tolerance of fruit to post-harvest mechanical injury (Keren-Keiserman *et al.*, 2004). In contrast, skin reticulation is not typically a quality concern for cucumber since fruit are consumed at immature stages (Zhang *et al.*, 2022). Nevertheless, studies in both crops showed that *SHN1* gene homologues (*CmSHN1* and *CsSHN1*), displaying high exocarp expression, serve as strong candidate genes for netting density traits (Oren *et al.*, 2020; Zhang *et al.*, 2022). For fleshy fruit crops, russeting is more strongly associated with poorer commercial quality (Faust and Shear, 1972). In apple, the MdSHN3 TF (Lashbrooke *et al.*, 2015b) and MdABCG12 transporter (Falginella *et al*., 2015) have both been positively linked to proper cuticle formation, and the absence of russet (Fig. 2G, H). In addition, the tomato DEFECTIVE IN CUTICULAR RIDGES (DCR) enzyme is required for cutin deposition, with silenced lines showing significant reductions in cutin content and subsequent cuticle failure (Fig. 2I; Lashbrooke *et al.*, 2016). This, in turn, resulted in the suberization of the tomato fruit in a process found to be transcriptionally analogous to apple russet, seed coat formation, and root suberization.

Mild cuticle failure can also predispose fruit to post-harvest dehydration and pathogen infection, thus resulting in crop loss. For instance, *SlSHN3* and its downstream targets, *SlMIXTA-like* and *SlCYP86A69*, have been shown to promote cuticle development and the resistance of tomato fruit to *Colletotrichum coccodes* infection and water loss (Fig. 2I; Shi *et al.*, 2013; Lashbrooke *et al.*, 2015a). Similar phenotypes have likewise been observed for the *CUTIN DEFICIENT1* (*CD1*)/*SlGDSL1* and *CD2* genes (Fig. 2I; Isaacson *et al.*, 2009; Girard *et al.*, 2012; Yeats *et al.*, 2012). In cucumber, fruit overexpressing *CsWAX2* showed reduced *Botrytis cinerea* fungal decay and desiccation (Wang *et al.*, 2015a). Glossiness, over dull-appearing fruit, is another important post-harvest quality trait in cucumber production. The glossy phenotype has been linked to the function of the *CsWIN1* (Zhang *et al.*, 2019) and trichome-related *CsTu*/*DULL* (Zhai *et al.*, 2022) genes, which are positively correlated with wax deposition in the fruit peel (Box 1).

Box 1. **The inter-related nature of crop surface development**

**Fig. 3.**
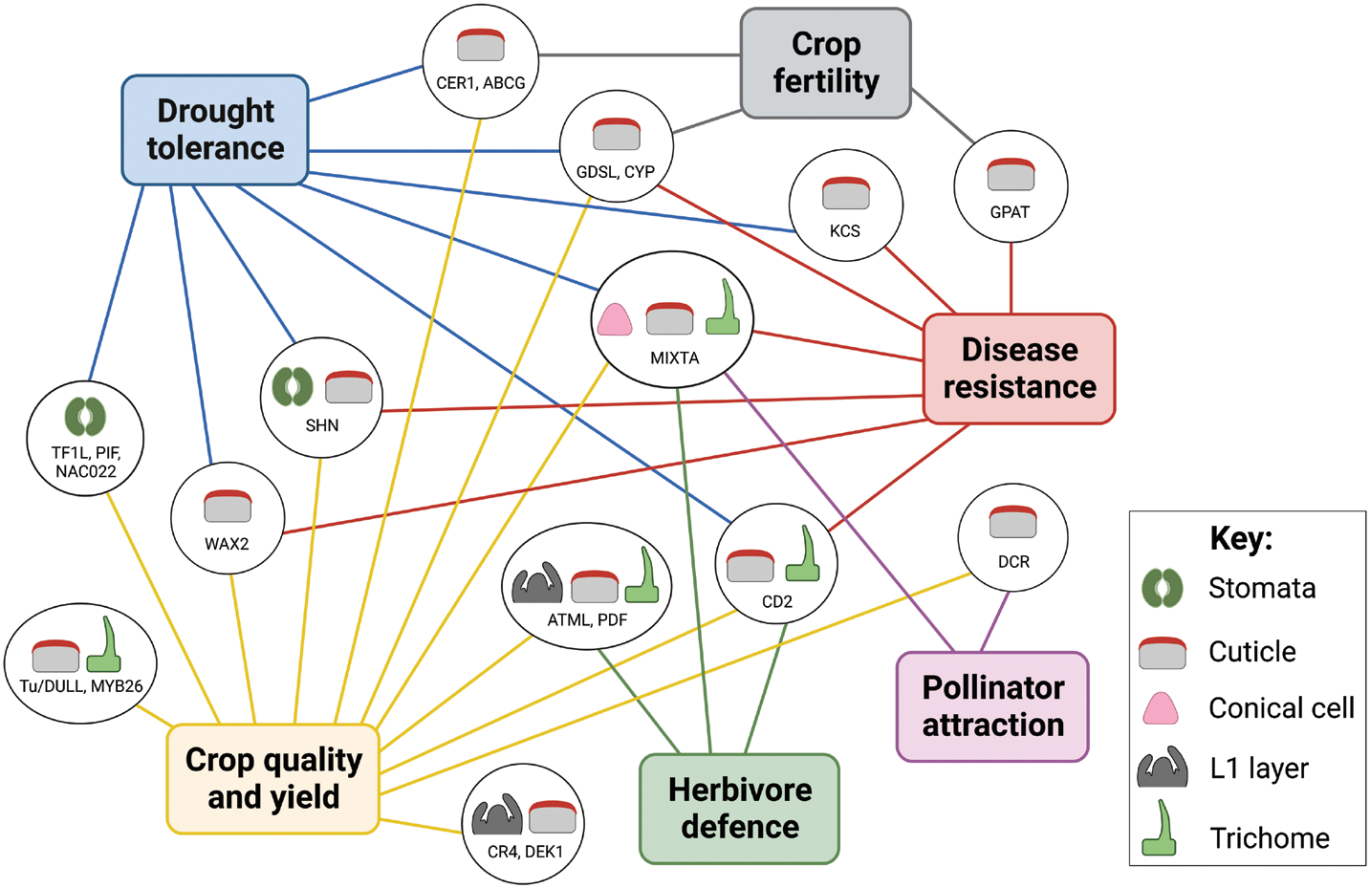
Network illustrating the interconnected nature of different surface elements (stomata, cuticle, conical cell, L1 layer, and trichome), driven by various crop genes, and their connection to beneficial traits (drought tolerance, crop fertility, disease resistance, pollinator attraction, herbivore defence, as well as crop quality and yield). Crop genes were selected based on their characterized involvement in the formation of two or more surface elements and/or their contributions towards two or more targeted traits.

Since the plant cuticle and epidermal cells both arise from the L1 tissue, it is expected that crosstalk between their regulatory pathways exists. This would be essential during surface differentiation to ensure the development of a functional outer layer. However, the nature of these interactions is poorly understood. Homologues of the SHN and MIXTA TFs, which form part of the same regulatory network (Shi *et al.*, 2013), serve as useful examples illustrating the interconnection between different surface elements and traits (Fig. 3). For instance, SHN proteins directly regulate cuticle biosynthetic enzymes (Kannangara *et al.*, 2007); however, their overexpression has also led to reduced stomatal densities which, combined, contribute to drought tolerance (Aharoni *et al.*, 2004; Bi *et al.*, 2018). Although the down-regulation of well-known stomatal genes was reported (J. Yang *et al.*, 2011), direct links between SHN and stomatal formation have not yet been made. Thus, it can be speculated that the increased wax load in overexpressing lines may indirectly impact stomatal formation as a compensatory response. Additional examples include the *MIXTA*-related genes of peach (*Prunus persica*) and cucumber. Interestingly, a loss-of-function mutation in the *PpeMYB25* gene led to a lack of peach fuzz (trichomes), resulting in the distinctive glabrous nectarine variety (Vendramin *et al.*, 2014). Recent studies have also illustrated that the shinier surfaces of nectarine fruit are, in part, due to the inability of the non-functional PpeMYB25 TF to activate *PpeMYB26* expression, a gene involved in wax accumulation and fruit dullness (Yang *et al.*, 2022). Similarly, a cucumber MIXTA orthologue, CsMYB6, has been shown to directly interact with CsTu/DULL (L. Zhao *et al.*, 2020), an important protein linked to tubercule initiation and wax deposition in the fruit peel, resulting in glabrous and shiny fruit surfaces in cucumber mutants (Yang *et al.*, 2014; Zhai *et al.*, 2022). These few studies alone illustrate the close connection between different surface structures, and also demonstrate the need to better understand their complex interactions to be able to fine-tune surface manipulation for crop improvement.

## Drought tolerance

### Stomata-related water-use efficiency

Drought and water stress tolerance have been associated with stomatal density and aperture in a variety of crop species, based on the impact of stomatal functionality on WUE. Despite this trait benefit, one of the major concerns in altering stomatal features to improve WUE is the detrimental impact that reduced stomatal function can have on the photosynthetic capacity and, subsequently, the biomass of crops. In addition, reducing transpirational water loss in crops can impact their evaporative cooling ability, leading to issues with heat stress and fertility (Bertolino *et al*., 2019). Nevertheless, genes involved in stomatal formation (Supplementary Table S1) serve as promising targets for the development of crops better suited to drought-prone areas, should these trade-off phenotypes prove to be negligible.

One approach that can be used to alter stomatal function is to hinder the development of functional stomatal complexes or to limit the number of cells entering the stomatal lineage (i.e. stomatal density). Studies aimed at characterizing genes involved in stomatal formation, however, are extremely limited for dicot crops. In the case of tomato, the *SPEECHLESS* (*SPCH*), *MUTE*, and *FAMA* gene orthologues, involved in stomatal differentiation, were shown to complement their respective Arabidopsis mutants (Ortega *et al.*, 2019). In addition, Morales-Navarro *et al.* (2018) found that the wild tomato (*Solanum chilense*) orthologue of the *STOMATAL DENSITY AND DISTRIBUTION1* gene, *SchSDD1-like*, was responsible for a significant reduction in stomatal density and an improved dehydration avoidance in overexpressing cultivated tomato. Similar observations were found for soybean (*Glycine max*), where *GmSPCH1* was able to rescue the stomatal phenotype of *atspch-3* mutants (Danzer *et al.*, 2015). Danzer *et al.* (2015) also reported that the collective down-regulation of four paralogous genes (*GmSPCH1–GmSPCH4*) led to the development of leaf surfaces devoid of stomatal and meristemoid complexes. In comparison, significant progress has been made in establishing key regulators of stomatal formation in monocot crops, which show fundamental deviated functions compared with dicots (Box 2).

Box 2. **Functional divergence in key regulators of stomatal differentiation in monocots versus dicots**

Based on their morphological and spatial differences, it is unsurprising that the regulators of monocot stomatal lineage cells have acquired diverged functions to those in dicots. This is due to not only variances in guard cell shape (e.g. dumbbell-shaped versus kidney-shaped) or stomatal distribution (e.g. linear file versus scatter-type), but also the additional formation of subsidiary cells (SCs) in monocot varieties (Conklin *et al.*, 2019; Buckley *et al.*, 2020). For example, the *OsFAMA* gene, which is functionally interchangeable with *AtFAMA*, holds an additional role in controlling the cell fate transition from subsidiary mother cells (SMCs) to SCs, as well as SMC division in rice (Liu *et al.*, 2009; Wu *et al.*, 2019). Further, rice and maize *MUTE* genes cause arrested meristemoids with a lack of SCs in loss-of-function mutants, indicating an important role in GMC fate transition, along with a deviated function in SC recruitment (H. Wang *et al.*, 2019; Wu *et al.*, 2019). The rice FOUR LIPS (OsFLP) protein has been linked to the control of GMC division orientation, rather than its timing (Lai *et al.*, 2005; Wu *et al.*, 2019), while gene knockout of *OsSDD1* resulted in the typical stomatal clustering phenotype observed in Arabidopsis, but with no impact on stomatal file density (Yu *et al.*, 2020). Interestingly, the SCARECROW (SCR; Di Laurenzio *et al.*, 1996) and SHORT ROOT (SR; Helariutta *et al.*, 2000) TFs, which have previously only been connected to root endodermal cell specification in Arabidopsis, were shown to directly interact and positively influence stomatal initiation and SC formation in rice (Wu *et al.*, 2019). There is, however, the conservation of function of the OsSPCH1 and OsSPCH2 proteins, which act redundantly in regulating the entry division of stomatal lineage cells (Wu *et al.*, 2019), while rice orthologues of the SCREAM1 (OsICE1) and SCRM2 (OsICE2) TFs were found to interact with OsSPCH1/2, OsMUTE and OsFAMA in a manner comparable with that observed in Arabidopsis (Kanaoka *et al.*, 2008; Wu *et al.*, 2019).

Further studies have assayed for changes in the drought tolerance of monocot crops, based on stomatal density manipulation. EPIDERMAL PATTERNING FACTOR (EPF)-related signalling proteins, when overexpressed, cause arrested meristemoid and guard mother cell (GMC) phenotypes in barley (Hughes *et al.*, 2017), rice (Caine *et al.*, 2019), and wheat (*Triticum aestivum*) (Dunn *et al.*, 2019), and result in an enhanced WUE without yield loss. Lu *et al.* (2019) further found that *OsMUTE* and *OsFAMA* were significantly down-regulated in rice plants overexpressing *OsEPF1* and *OsEPF2*, while the severity of arrested stomatal phenotypes was greater for *OsEPF2*, consistent with an earlier role in negatively regulating stomatal lineage cell entry (Lee *et al.*, 2012). In contrast, rice *STOMAGEN/EPFL9* gene orthologues, *OsEPFL9-1* and *OsEPFL9-2*, were shown to positively regulate stomatal density in rice (Lu *et al.*, 2019), with similar phenotypes observed for the dicot *VviEPFL9-1* gene, in addition to an improved WUE in grapevine (*Vitis vinifera*) (Clemens *et al.*, 2022). The receptor-like kinase family member Salt-Inducible Kinase 1 (OsSIK1) has high sequence homology to the kinase domain of three Arabidopsis ERECTA family proteins, causing significant reductions in stomatal density and a higher tolerance to desiccation in overexpressing rice plants (Ouyang *et al.*, 2010).

Aside from stomatal formation, manipulating stomatal closure may improve the drought response of crops. In this regard, ABA and drought treatments were shown to reduce the expression of the guard cell-specific *AtMYB60* gene, driving stomatal closure and improving resilience to dehydration in null mutants (Cominelli *et al.*, 2005). Likewise, the grapevine *MYB60* gene orthologue (*VviMYB60*) is negatively regulated in response to ABA and functionally complements the *atmyb60-1* mutant, restoring proper stomatal function and confirming its role as a positive regulator in stomatal aperture (Galbiati *et al.*, 2011). Under drought stress, rice plants overexpressing the *OsTF1L* and *OsNAC022* genes show phenotypes of increased stomatal closure coupled with an enhanced drought tolerance, as well as increased grain yield in the case of *OsTF1L* (Hong *et al.*, 2016; Bang *et al.*, 2019). This was also observed for two ABA-inducible maize phytochrome-interacting factors (PIFs) basic helix–loop–helix (bHLH) TFs, ZmPIF1 and ZmPIF3, in overexpressing rice plants (Gao *et al.*, 2018a, b). Despite having positive effects on grain yield under drought stress, the reduced stomatal aperture observed in these transgenic rice lines proved to be detrimental to plant development under normal growth conditions. Thus, the outcome of stomatal manipulation can present with the issue of deciding which trade-off is most beneficial to crop production.

### Non-stomatal water loss

As an alternative, modifying the cuticular layer can assist in improving the tolerance of crops to water deficit by hindering non-stomatal water loss. This role is largely connected to the hydrophobic wax component rather than the cutin (Schreiber, 2010). Nevertheless, both wax and cutin biosynthetic enzymes, such as ECERIFERUM1 (CER1), CER3/WAX2, enoyl-CoA reductase (ECR), glycerol-3-phosphate acyltransferase (GPAT), β-ketoacyl-CoA synthase (KCS), β-ketoacyl-CoA reductase (KCR), and long-chain acyl-CoA synthetase (LACS) homologues, as well as cuticle transport and cutin assembly proteins [ATP-binding cassette (ABC) transporters and GDSL, respectively], have been shown to play a fundamental role in the drought tolerance of apple (Qi *et al.*, 2019; Lian *et al.*, 2021; Li *et al.*, 2022), barley (Chen *et al.*, 2011; Li *et al.*, 2017, 2018), cucumber (Wang *et al.*, 2015a, b), grapevine (Liu *et al.*, 2023), orange (*Citrus sinensis*) (D. Liu *et al.*, 2022), peanut (*Arachis hypogaea*) (Lokesh *et al.*, 2019), rice (Yu *et al.*, 2008; Islam *et al.*, 2009; Park *et al.*, 2010; Chen *et al.*, 2011; Qin *et al.*, 2011; Zhou *et al.*, 2013, 2015), tomato (Wu *et al.*, 2022), and wheat (Li *et al.*, 2019). OsCYP96B5, encoded by the *Wax crystal-Sparse Leaf 5* (*WSL5*) gene, also forms part of this pathway by catalysing the terminal hydroxylation of alkanes to C29 primary alcohols in rice (Zhang *et al.*, 2020). Zhang *et al.* (2020) found that *wsl5* mutant leaf cuticles displayed a significant increase and reduction in alkane and C29 primary alcohol content, respectively, leading to an improved drought response. β-Diketone contributes to the glaucous nature of wheat and barley varieties; a trait that has been associated with higher grain yields under drought conditions (Fleury *et al.*, 2010; Bi *et al.*, 2016). Three genes involved in β-diketone synthesis [*Diketone Metabolism-Polyketide synthase* (*DMP*), *DM-Hydrolase* (*DMH*), and *DM-CYP450* (*DMC*)] were identified and found to be positioned in conserved metabolic gene clusters present in the *W1* and syntenic *Cer-cqu* loci of wheat and barley, respectively (Hen-Avivi *et al.*, 2016; Schneider *et al.*, 2016). In wheat, *DMP*- and *DMH*-silenced plants displayed a glossy phenotype, with fewer epicuticular wax crystals present on the flag leaf sheaths and spikes, as well as significantly lower levels of β-diketone and its derivatives (Hen-Avivi *et al.*, 2016).

In terms of transcriptional regulation, AP2-domain SHINE (SHN) proteins were found to promote cuticle deposition and improve plant resilience to drought conditions in apple (Zhang *et al.*, 2019a), papaya (*Carica papaya*) (Girón-Ramírez *et al.*, 2021), rice (Wang *et al.*, 2012; Zhou *et al.*, 2014), sorghum (*Sorghum bicolor*) (Bao *et al.*, 2017), tomato (Al-Abdallat *et al.*, 2014), and wheat (Djemal and Khoudi, 2016; Bi *et al.*, 2018), however also increasing water loss rates in soybean (Xu *et al.*, 2016). For wheat, the observed drought tolerance in overexpressing lines was also, in part, due to a significant reduction in stomatal density (Bi *et al.*, 2018). Regarding R2R3-MYB proteins, the MIXTA-like SlMX1 TF, which acts upstream of SlSHN2, drives cuticle formation in tomato leaves and enhances plant recovery rates from water deficiency (Ewas *et al.*, 2016), while OsMYB60 promotes wax accumulation in rice by activating the expression of wax-related genes (e.g. *OsCER1*), thereby contributing to plant resistance against drought (Jian *et al.*, 2022). Lastly, ZmMYB94 activity, which is responsible for the *fused leaves1* maize mutant, has an important cuticle-related function in the separation of organs during development and is associated with the response of maize to drought stimuli (La Rocca *et al.*, 2015; Castorina *et al.*, 2020).

Proteins involved in epigenetic and post-transcriptional modulation of drought-related cuticle properties have been characterized in rice. Mutations in the *OsCHR4* gene, which encodes a member of the chromodomain helicase DNA-binding3 (CHD3) family involved in chromatin remodelling, resulted in the up-regulation of auxin and wax-related genes leading to altered leaf morphology and increased leaf cuticular waxes, respectively (Guo *et al.*, 2019). The greater amount of wax deposits found in the *oschr4-5* mutant leaves resulted in an amplified resistance to drought stress (Guo *et al.*, 2019). In terms of post-transcriptional regulation, the rice *DROUGHT HYPERSENSITIVE* (*DHS*) gene, encoding a Really Interesting New Gene (RING)-type E3 ligase, was found to be a negative regulator of wax biosynthesis, since its overexpression caused significant reductions in wax load and a hypersensitive response to drought (Wang *et al.*, 2018). DHS acts by promoting the degradation of RICE OUTERMOST CELL-SPECIFIC GENE4 (ROC4), an HD-ZIP IV family member that positively modulates cuticular wax formation through direct activation of *OsBODYGUARD* (*OsBDG*) expression (Wang *et al.*, 2018). In Arabidopsis, it has been speculated that BDG not only holds an indirect role in improving drought tolerance by maintaining cuticle integrity, but also acts as a mediator for osmotic stress induction of ABA biosynthesis (Z.Y. Wang *et al.*, 2011). While the mechanisms for cuticular regulation of ABA biosynthesis have not been elucidated further, this research holds great significance for crop species and agriculture, where the application of plant growth regulators such as ABA may impact surface-related traits.

## Biotic stress tolerance

### Herbivore defence

Tomato forms glandular leaf trichomes (type I, IV, and VI) which secrete metabolites that either repel or poison insects, as well as non-glandular leaf trichomes (types II, III, and V) that serve as physical barriers against herbivory (Tian *et al.*, 2012). Consequently, genes involved in leaf trichome formation in tomato are deemed useful targets for altering pest-related defence traits (Supplementary Table S1). In this regard, L1-related *Woolly* (*Wo*) has been identified as a key gene involved in trichome initiation, causing significant increases in glandular trichome (type IV) density in tomato mutant leaves during juvenile development (Vendemiatti *et al.*, 2017). In addition, Wo is predicted to regulate glandular trichome (type I) development by forming separate heterodimers with Hair (H), which is a single C2H2 zinc-finger protein (ZFP), as well as the B-type cyclin protein SlCycB2 (C. Yang *et al.*, 2011; Chang *et al.*, 2018). Further assays revealed that a close homologue of H (SlZFP8-like) dimerizes with both H and Wo, which act cooperatively to regulate trichome initiation and elongation, with SlZFP6 acting downstream (Zheng *et al.*, 2022). Interestingly, overexpressing *SlCycB2*, and its direct transcriptional activator *SlMYB75*, resulted in significantly fewer glandular leaf trichomes, lower terpene content, and an increased susceptibility to *Prodenia litura* and spider mite herbivory, respectively (Gao *et al.*, 2017; Gong *et al.*, 2021). SlMIXTA-like proteins have also been identified as negative regulators of trichome initiation, with silenced and knockout tomato lines displaying higher leaf trichome densities and clustering patterns (Galdon-Armero *et al.*, 2020). In terms of positive modulators, mutations in the cutin-related *SlCD2* and bHLH-encoding *SlMYC1* genes cause a significant reduction and absence of vegetative type VI trichomes, respectively, as well as lower the trichome-related terpene content of *cd2* mutant leaves (Nadakuduti *et al.*, 2012; Xu *et al.*, 2018). The proper morphogenesis of tomato trichomes is essential for their ability to synthesize defence metabolites and protect against pests (Kang *et al.*, 2016). This was evident in two tomato mutants which displayed altered trichome shapes connected to defects in actin filament polymerization. The tomato *hairless* (*hl*) mutant caused bending and swelling of type VI trichomes which led to an impaired synthesis of terpene compounds and a decreased resistance to *Manduca sexta* feeding (Kang *et al.*, 2016), while the *inquieta* (*ini*) mutant displayed similar morphological phenotypes (Jeong *et al.*, 2017). The genes responsible for these mutant phenotypes, *SRA1* (*hl*) and *ARPC2A* (*ini*), encode components of the SCAR/WAVE and ARP2/3 complexes, respectively, which have previously been linked to trichome morphogenesis in Arabidopsis (Basu *et al.*, 2004; El-Din El-Assal *et al.*, 2004). Recent studies have shown that SRA1 may interact with Hairless-2 (Hl-2), a NAP1 subunit of the SCAR/WAVE complex, to drive normal trichome development, while *Hl*-*2* expression is also modulated by an HD-ZIP IV protein (SlHDZIV8; Q. Xie *et al.*, 2020). Several hormone signalling proteins have been linked to the formation of multicellular trichomes in tomato (refer to the review of Chalvin *et al.*, 2020).

### Disease resistance

Cutin and cuticular waxes play key roles in plant–pathogen interactions and, as a result, disease development in crops. The CHD3-type chromatin-remodelling factor TaCHR729, which is recruited by TaKCS6 promoter-associated bHLH-type transcription factor1 (TaKPAB1), was found to repress *TaKCS6* expression and lower the conidial germination rate of *Blumeria graminis* f.sp. *tritici* (*Bgt*) on silenced wheat leaves displaying reduced wax loads (Wang *et al.*, 2019). Wang *et al.* (2019) further discovered that *Bgt* conidial germination was in fact stimulated by the presence of wax-related very long chain (VLC) aldehydes. Similar phenotypes were observed for the wax-related TaECR enzyme (Kong *et al.*, 2020), as well as for *HvKCS1* and *HvKCS6* loss-of-function barley mutants (Weidenbach *et al.*, 2014; Li *et al.*, 2018). In barley, the WIN1 TF was found to be positively associated with an enhanced tolerance to Fusarium head blight (*Fusarium graminearum*) (Kumar *et al.*, 2016). Constitutive expression of the stress-responsive apple *MYB30* gene altered the structural and compositional properties of vegetative Arabidopsis cuticles, while improving resistance to *Botryosphaeria dothidea* in apple calli (Zhang *et al.*, 2019b). However, cuticle–pathogen interactions are complex and, in many instances, opposing responses can be observed depending on the pathogen studied. This is evident in the case of the loss-of-function mutant of the cutin-related tomato GPAT6 enzyme, which exacerbated susceptibility to *Phytophthora* pathogens, but improved resistance to *B. cinerea* (Fawke *et al.*, 2019).

Secondary metabolites such as triterpenoids are known to have diverse plant functions including defence against plant pathogens (Smaili *et al.*, 2019). Targeting pathways that promote triterpenoid production may aid in improving crop resistance to diseases; however, studies associating these two factors are restricted. During their biosynthesis, the first step in the diversification of triterpene compounds involves the cyclization of 2,3-oxidosqualene via oxidosqualene cyclases (OSCs). Genes encoding OSCs have been linked to the production of cuticle-related triterpenes in apple (Andre *et al.*, 2016), sweet wormwood (*Artemisia annua*; Moses *et al.*, 2015), and tomato (Z. Wang *et al.*, 2011) (Supplementary Table S1). In the case of apple and sweet wormwood, further triterpene C-28 and C-3 diversification, respectively, was shown to require the additional oxidative activity of CYP716 enzymes. In grapevine, the triterpene-related VviMYB5b TF, which is highly expressed in berry skins (Deluc *et al.*, 2008), proved to have an important function in several biological processes including the negative modulation of triterpenoid accumulation in overexpressing tomato lines (Mahjoub *et al.*, 2009).

### Pollinator attraction

The conical shape of petal epidermal cells contributes to their colour intensity and brightness, thereby impacting pollinator attraction and seed-setting rates (Glover and Martin, 1998). In the case of tomato, the cuticle and trichome-related SlMIXTA-like protein has also been linked to the positive modulation of conical cell patterning on petal surfaces (Galdon-Armero *et al.*, 2020). This role has likewise been observed for the *MIXTA-like* homologue of orchid (*Phalaenopsis*; Lu *et al.*, 2022), as well as those of petunia (*Petunia hybrida*) and snapdragon (*Antirrhinum majus*) which negatively impact the presentation and architecture of flowers in null mutants (Baumann *et al.*, 2007).

Another epidermal feature that may contribute to pollinator attraction is the presence of nanoridges (cuticular folds) on petal surfaces. Nanoridges are speculated to assist in pollinator attraction and attachment by influencing the light refractive and tactile properties of flowers, respectively (Koch *et al.*, 2008; Whitney *et al.*, 2009). An AP2/ERF Va2 subgroup member (PeERF1) has been positively associated with nanoridge formation in orchid floral epidermal lips during late stages of morphogenesis (Lai *et al.*, 2020). In addition, silenced orchid lines showed reduced expression of cuticle-related *PeDCR*, *PeCYP77A4*, and *PeGPAT* genes which have previously been connected to nanoridge formation in Arabidopsis petals (Li-Beisson *et al.*, 2009; Panikashvili *et al.*, 2009; Mazurek *et al.*, 2017).

### Crop fertility

Genes involved in anther cuticle formation have been linked to male sterility in cereal crops, a beneficial trait for hybrid crop breeding (Supplementary Table S1). This is due to the dehydration and subsequent deformation of anthers in mutant or knockdown lines, which has been illustrated for many cuticle biosynthetic enzymes in maize (Somaratne *et al.*, 2017; Xie *et al.*, 2018; An *et al.*, 2019; Jiang *et al.*, 2021; S. Zhang *et al.*, 2021), and rice (Jung *et al.*, 2006; Men *et al.*, 2017; Xu *et al.*, 2017; J. Zhao *et al.*, 2020). In addition, OsABCG26 and ZmABCG26 are suggested to drive the export of cutin from the tapetal cells to the anther surfaces in rice and maize, respectively, causing significant reductions in anther cuticle deposition in male-sterile knockout mutants (Zhao *et al.*, 2015; Jiang *et al.*, 2021).

## Biotechnological strategies for improving crop surface traits

Since new biotechnology methods and tools have been developed in the past few years, we briefly introduce the latest techniques and how they could be applied in the future for surface-based crop improvement. For non-biotech breeding, we direct the reader to the comprehensive review of Petit *et al.* (2017). Classical breeding and novel genomic technologies, however, are not antagonistic, and their integration could increase the speed and precision of breeding programmes, as has recently been reported (Lyzenga *et al*., 2021).

### Transgenesis and cisgenesis/intragenesis

**Fig. 4.**
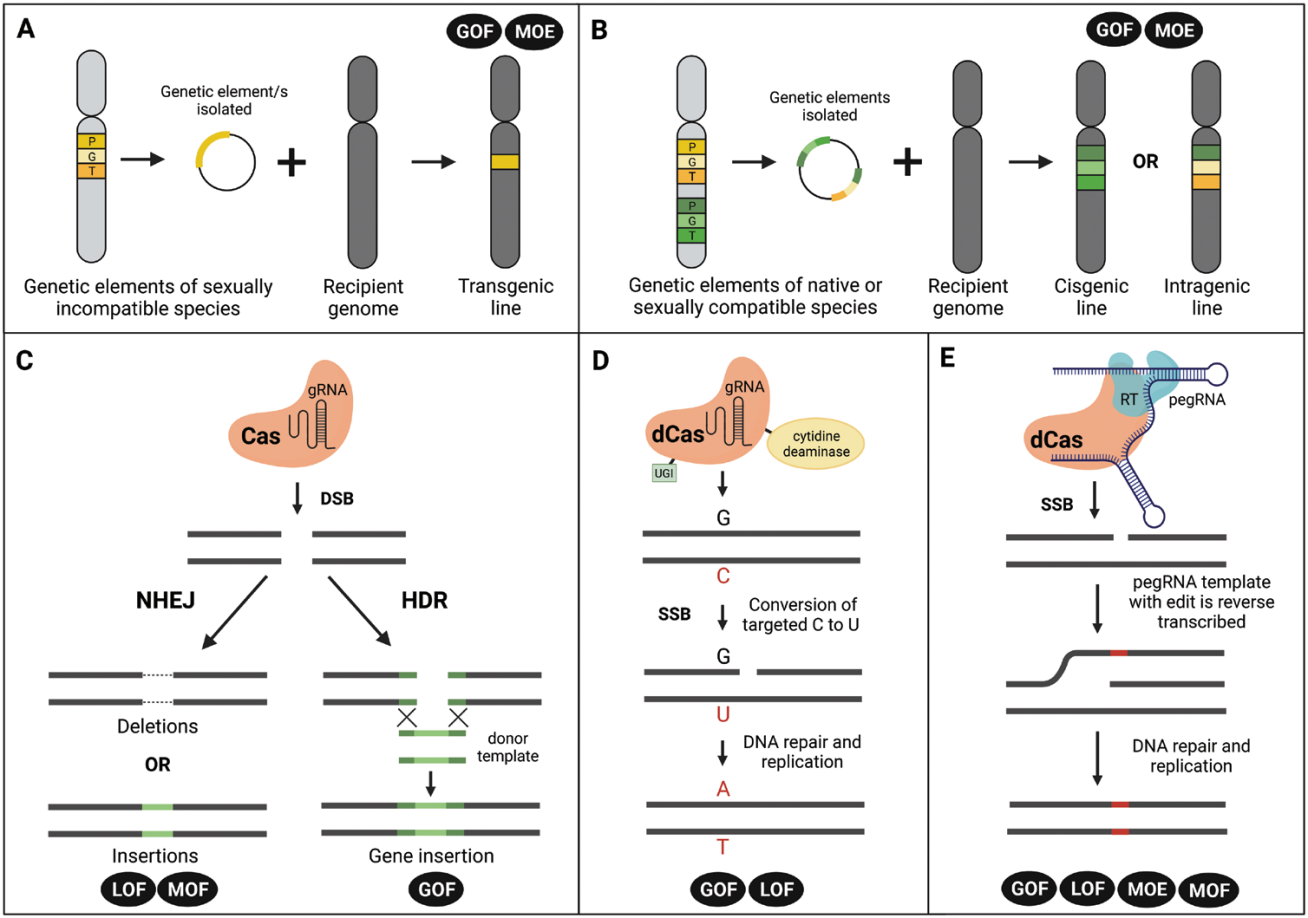
Schematic illustration of genetic engineering techniques available for crop gene modification. (A) Transgenesis, involving the incorporation of genetic elements, derived from a sexually incompatible species, into a crop genome of interest. (B) Cisgenesis/intragenesis, involving the incorporation of genetic elements, derived from sexually compatible or native species, into a crop genome of interest. (C) DSB-mediated editing, with the assistance of target-specific nucleases (e.g. Cas engineered with a guide RNA), induces the NHEJ and HDR cell DNA repair mechanisms. For NHEJ, this leads to random insertions and deletions, while HDR, with the presence of a donor template, can insert or replace genes. DSB, double-strand break; NHEJ, non-homologous end joining; HDR, homology-directed repair. (D) Base editing requires a catalytically inactive nuclease (e.g. dCas engineered with a guide RNA) that is fused to a deaminase protein. The example provided illustrates the activity of a CBE which, in the presence of UGI, drives the deamination of cytosine to uracil. SSBs in the non-targeted strand stimulate DNA repair mechanisms which, following replication, leads to a C-to-T base substitution. CBE, cytosine base editor; UGI, uracil glycosylase inhibitor; SSB, single-strand break. (E) Prime editing requires a catalytically inactive nuclease (e.g. dCas engineered with a pegRNA, prime editing guide RNA) that is fused to a reverse transcriptase. Prime editors induce SSBs on the non-targeted strand to prime reverse transcription of the pegRNA template (containing the edit). During DNA repair and replication, the edited strand is integrated and copied into the complementary strand. Types of gene modification that can be achieved by each GE tool are highlighted by black boxes and include gain of function (GOF), loss of function (LOF), modulation of expression (MOE), and modulation of function (MOF).

Transgenesis is highly useful within crop production since it allows for the transfer of genetic elements between species that are sexually incompatible (Fig. 4A). This provides the ability to incorporate genetic diversity that would otherwise be impossible to achieve through traditional breeding, and accelerate crop improvement. An extensive list of genes characterized for their involvement in crop surface traits is given in Supplementary Table S1. Genes encoding enzymes involved in metabolic pathways forming the constituents of the cuticular layer are strong candidates for a transgenic approach due to the massive diversity seen in these metabolites across plant species. For instance, heterologous expression of *OSC* genes or modifying *CYP450* genes may allow for the production of diverse triterpenoids in crops which are unable to accumulate these compounds naturally. Tens of thousands of bioactive triterpenoids exist across the plant kingdom, yet in domesticated tomato and apple fewer than a dozen are present (Fernandez-Moreno *et al*., 2016). Thus, metabolic engineering of these pathways into domesticated crops has the potential to improve pathogen resistance and nutritional value. Further, heterologous expression of genes coding for enzymes that modify VLC fatty acids, found in cuticular waxes, can result in the production of specialized waxes not typical of a selected crop. The *W1* locus of wheat is an interesting candidate in this regard, since it could be expressed in other crops to produce cuticular β-diketone. One of the major limitations in creating transgenic crop varieties, however, is the general lack of public acceptance surrounding genetic modification (Lassen *et al*., 2002).

In response to this, two alternative transformation concepts have been developed, namely cisgenesis and intragenesis. Both approaches integrate genetic elements that are sourced only from the native species or species that are cross-compatible. For cisgenesis, the gene of interest is integrated with its native promoter and terminator (Lusser and Davies, 2013), while intragenesis refers to the hybridization of genetic elements taken from different genes and loci (Fig. 4B; Limera *et al.*, 2017). The gene pool available for cisgenesis and intragenesis is thus identical to that of conventional breeding; however, results are achieved within a shorter time and bypass the potential carryover of negative traits due to linkage drag (Holme *et al.*, 2013). Currently, the most common application of cisgenic and intragenic strategies has been for the gain of function and expression modulation of crop genes in the context of improving pathogen resistance and quality traits (reviewed in Holme *et al*., 2013; Giudice *et al.*, 2021). With regards to crop surface improvement, a strong candidate for these approaches is the overexpression of *SHN* orthologues. Since *SHN* orthologues have been functionally characterized in several crops, where they positively regulate cuticle formation (Supplementary Table S1), it is likely that a native orthologue of this gene exists in the crop species of interest. This negates the need for transgenesis and, provided suitable native promoters can be identified, increasing the expression of native *SHN* orthologues will foreseeably lead to increased cuticle formation along with its concomitant benefits. Another target that may be well suited for cisgenic crop improvement is the *ABCG12* gene. Evidence suggests that expression of a functional allele of this gene in apple will prevent the occurrence of fruit skin russeting (Falginella *et al*., 2015). Finally, improvement of cotton fibre quality could probably be achieved through the cisgenic expression of key positive regulators of lint fibre development, such as the *GhHOX3* and *GhMML4* genes (Shan *et al.*, 2014; Wu *et al.*, 2018).

In spite of having favourable outputs, all three biotechnology applications have the same limitation in that, with current modes of transformation, gene integration occurs randomly within the crop genome and may result in multiple copies, potentially leading to the generation of crop lines with off-target trait effects (Basso *et al.*, 2020).

### Gene editing technologies

Alternatively, gene editing (GE) technologies allow for the introduction of genetic mutations in a more precise and efficient manner. In particular, by means of an RNA-guided clustered regularly interspaced palindromic repeats (CRISPR)-associated protein (Cas) nuclease which has gained significant popularity in recent years due to its high precision, design simplicity, and cost effectiveness (Ghogare *et al*., 2020). Currently, there are three main techniques available for targeted mutagenesis, gene replacement, or deletion using these nucleases. These comprise gene modifications arising from double-strand breaks (DSBs), base editing, or prime editing. Target-specific DSBs, driven by engineered Cas nucleases, activate the two main cell DNA repair pathways, namely non-homologous end joining (NHEJ) and homology-directed repair (HDR) (Fig. 4C). NHEJ is prone to errors, resulting in random insertions or deletions at the target site. If the sequence modification causes a frameshift or alters key amino acid residues in the final gene product, this can lead to the loss of function or modulation of function of a gene, respectively (Voytas and Gao, 2014). Indeed, most gene editing that has contributed to crop improvement thus far has been through loss-of-function mutations generated by NHEJ-mediated repair (Ahmad *et al.*, 2021). Interesting surface targets for this approach include stomatal formation and aperture-regulating genes, such as *SPCH*, *EPFL9*, and *MYB60* (Supplementary Table S1). Reducing the expression of these genes has been shown to produce favourable water stress response phenotypes in several crops including grapevine, rice, soybean, and tomato (Galbiati *et al.*, 2011; Danzer *et al.*, 2015; Ortega *et al.*, 2019; Clemens *et al*., 2022). The fact that orthologues appear to play a similar role in both monocots and dicots suggests that generating loss-of-function mutations for orthologues in a crop of choice will be a successful strategy for improving crop WUE. When altering stomatal formation and aperture, through induced mutations in the above-mentioned genes, one should consider the potential effect on plant physiology and yield, as modulating water availability will have direct consequences for plant growth. Besides agricultural traits, this strategy can also be implemented for consumer-based fruit quality attributes, such as pigmentation. Gene editing disruption of the tomato *MYB12* or *CHS* gene is a potential strategy for the generation of pink-coloured tomatoes (due to the absence of the yellow naringenin chalcone pigment), which are prioritized in Asian markets and are increasing in popularity worldwide (Ballester *et al.*, 2010). Disrupting the function of a crop gene is not always beneficial, however, and the introduction of a functional gene/allele may be required. In this regard, a more useful alternative is the HDR pathway, which provides the possibility of site-directed gene insertion/replacement. Despite this benefit, its application in crop improvement has been limited due to the infrequency of HDR within somatic plants cells and the inefficient delivery of donor templates (Azameti and Dauda, 2021; Chen *et al.*, 2022).

Base editing enables the transition or transversion of individual bases, resulting in the gain or loss of function of targeted crop genes (Li *et al.*, 2023). This is particularly useful when considering that many agronomically significant alleles, generated through conventional breeding, are caused by one or many single nucleotide polymorphisms (Chen *et al.*, 2022). Base editors comprise a catalytically inactive Cas nuclease (and guide RNA) with a cytidine or adenosine deaminase driving C-to-T or A-to-G base conversions, respectively, without generating DSBs (Fig. 4D; Azameti and Dauda, 2021). For cytosine base editors (CBEs), the additional activity of a uracil glycosylase inhibitor (UGI) is required to inhibit the base excision repair (BER) pathway and enhance the efficiency of base substitution (Komor *et al.*, 2016). Alternatively, studies have replaced the UGI with UG to promote the BER pathway and enable C-to-G base transversions (Li *et al.*, 2023). Studies that have successfully implemented base editing in crops are mostly restricted to monocots, with focus placed on improving herbicide tolerance, defence responses, and yield traits (Mishra *et al.*, 2020; Li *et al.*, 2023). Unfortunately, the variety of base modifications that can be achieved by current base editors is limited. In addition, restrictive protospacer adjacent motif (PAM) requirements and narrow editing windows impact their efficiency and specificity (Mishra *et al.*, 2020).

Bypassing these issues is the recent introduction of a search-and-replace GE tool known as prime editing. Prime editing is highly versatile, enabling any type of target-specific small insertions/deletions, replacements, and base modifications (Anzalone *et al.*, 2019). As such, it holds great potential as a method for achieving each gene modification approach. Prime editors consist of a catalytically inactive Cas nuclease programmed with a prime editing guide RNA (pegRNA) and reverse transcriptase which, together, identify the target site and encode the desired gene alteration (Fig. 4E; Anzalone *et al.*, 2019). However, prime editing in crops is still in its infancy, and significant progress needs to be made in optimizing the efficiency of its application (Hua *et al.*, 2022). Nevertheless, with the rapid evolution of GE techniques witnessed in recent years, we are confident that prime editing will become more efficient and applicable to a wide range of crops in the near future.

## Conclusion

The plant cuticle and epidermal structures form part of a complex multifunctional surface layer driven by a tightly regulated and interconnected gene network. In recent years, significant progress has been made in characterizing important surface-related genes connected to economically significant traits in a multitude of crops. While gaps are still evident in understanding their regulatory pathways, current research has provided a strong resource of possible candidate genes that can be modified for crop improvement. In addition, recent advancements in GE technologies (e.g. prime editing) have paved the way for the potential application of high precision targeted gene modifications of any type in a crop of interest. Combined, biotechnology-mediated breeding holds significant potential for the generation of crops with desirable surface traits impacting key agricultural parameters, such as improved commercial quality and drought tolerance. However, fundamental limitations linked to the possibility of pleiotropic and off-target effects, along with the recalcitrant nature of many crop species to regeneration during transformation, are just a few of the hurdles that biotechnology applications need to overcome.

## Supplementary data

The following supplementary data are available at *JXB* online.

Table S1. List of crop genes associated with above-ground cuticle and specialized epidermal cell formation.

erad321_suppl_Supplementary_Table_S1
