# Supplementary material for: Beyond skin-deep: targeting the plant surface for crop improvement
Source: J Exp Bot. 2023 Aug 17;74(21):6468–86. doi: 10.1093/jxb/erad321 (PMC10662250; doi:10.1093/jxb/erad321)
Supplement: erad321_suppl_Supplementary_Table_S1 [file erad321_suppl_supplementary_table_s1.pdf]

Jolliffe *et al.* (2023). Beyond skin-deep: targeting the plant surface for crop improvement. Journal of Experimental Botany.

Supplementary Table S1: List of crop genes associated with aboveground cuticle and specialised epidermal cell formation.

Gene*	General Surface Function	Protein Type	Native Species	Biotech Approach	Host Species	Phenotype	Potential Trait Target	Reference
CR4	L1 identity and cuticle formation	Receptor-like kinase	Rice	Transgenic (RNAi)<br>-	Rice<br>-	- Separated palea and lemma due to irregular epidermis.<br>- Impaired grain quality.<br>- Reduced grain size with wrinkled surface.<br>- Thinner discontinuous cuticle at palea-lemma interlock.	Crop quality and yield	Pu *et al.* (2012)<br>Chun *et al.* (2020)
ADL1	L1 identity	Calpain-like protease	Rice	-	-	- Abaxial bulliform-like cells and leaf rolling.<br>- Semi-stunted plant growth.	Crop quality and yield	Hibara *et al.* (2009)
CFL1	L1 identity and cuticle formation	WW-domain TF	Rice	Transgenic (OE)	Rice	- Curly leaves with defective cuticles.	Crop quality and yield	Wu *et al.* (2011)
ROC5	L1 identity	HD-ZIP IV TF	Rice	Transgenic (KO)	Rice	- Abaxial leaf rolling.<br>- Increased number and size of bulliform cells.	Crop quality and yield	Zou *et al.* (2011)
CR4	L1 identity and cuticle formation	Receptor-like kinase	Maize	-	-	- Crinkled and fused leaves.<br>- Irregular epidermis and cuticle deposition.<br>- Mosaic aleurone cells.<br>- Stunted plant growth	Crop quality and yield	Becraft *et al.* (1996), Jin *et al.* (2000)
DEK1	L1 identity	Calpain-like protease	Maize	-	-	- Crinkled leaves.<br>- Irregular epidermal cells.<br>- Mosaic aleurone cell formation.	Crop quality and yield	Becraft *et al.* (2002)
Nud	Cuticle formation	AP2/ERF-domain TF	Barley	-	-	- Loose hulls from lack of lipids at hull-caryopsis adhesion.	Crop quality and yield	Taketa *et al.* (2008)
WOX3B	Trichome formation	WUSCHEL-like HOX TF	Rice	Transgenic (RNAi)	Rice	- Glabrous leaves and hulls.<br>- Increased grain packing efficiency.	Crop quality and yield	Zhang *et al.* (2012)
HL6	Trichome formation	AP2/ERF-domain TF	Rice	Transgenic (OE/KO)<br>Transgenic (OE)	Rice	- Increased macrohair length (OE) and number (KO) on hull.<br>- Increased macrohair initiation on leaves.	Crop quality and yield	Sun *et al.* (2017)<br>Y. Xie *et al.* (2020)

WIN1	Cuticle formation	AP2/ERF-domain TF	Rapeseed	Transgenic (OE)	Rapeseed	- Increased leaf wax under salt stress.<br>- Increased seed oil content.	Crop quality and yield	Liu *et al.* (2019)
MYB2	Trichome formation	R2R3-MYB TF	Cotton	Transgenic (OE)<br><br>Transgenic (Complement)	Arabidopsis	- Induced seed trichome formation.<br>- Rescued trichome formation in *gl1* mutants.	Crop quality and yield	Wang *et al.* (2004)
MYB3	Trichome formation	R2R3-MYB TF	Cotton	Transgenic (Complement)	Arabidopsis	- Rescued trichome formation in *gl1* mutants.	Crop quality and yield	Shangguan *et al.* (2021)
TTG1/3	Trichome formation	WD40-repeat TF	Cotton	Transgenic (Complement)	Arabidopsis	- Rescued trichome formation in *ttg1* mutants.	Crop quality and yield	Humphries *et al.* (2005)
DEL65	Trichome formation	bHLH TF	Cotton	Transgenic (Complement)	Arabidopsis	- Partly rescued trichome formation in *gl3 egl3* mutants.	Crop quality and yield	Shangguan *et al.* (2016)
HOX1	Trichome formation	HD-ZIP IV TF	Cotton	Transgenic (Complement)	Arabidopsis	- Rescued trichome formation in *gl2-2* mutants.	Crop quality and yield	Guan *et al.* (2008)
HOX3	Trichome formation	HD-ZIP IV TF	Cotton	Transgenic (OE/RNAi)	Cotton	- Increased/decreased lint fibre length.	Crop quality and yield	Shan *et al.* (2014)
CPC	Trichome formation	R3-MYB TF	Cotton	Transgenic (OE)	Cotton	- Delayed fibre initiation and reduced fibre length.	Crop quality and yield	Liu *et al.*, (2015)
HD-1	L1 identity and trichome formation	HD-ZIP IV TF	Cotton	Transgenic (OE/RNAi)	Cotton	- Increased/delayed fibre initiation.<br>- Increased fuzz fibre (OE).	Crop quality and yield	Walford *et al.* (2012)
ML1	L1 identity and trichome formation	HD-ZIP IV TF	Cotton	Transgenic (OE)	Arabidopsis	- Increased stem and leaf trichomes.	Crop quality and yield	Zhang *et al.* (2010)
PDF1	L1 identity and trichome formation	HD-ZIP IV TF	Cotton	Transgenic (RNAi)	Cotton	- Delayed fibre initiation.<br>- Shorter fibres with reduce lint percentage.	Crop quality and yield	Deng *et al.* (2012)
MYB25	Trichome formation	R2R3-MYB TF	Cotton	Transgenic (OE/RNAi)	Cotton	- Increased fibre initiation (OE) and shorter fibres (RNAi).	Crop quality and yield	Machado *et al.* (2009)
MYB25-like	Trichome formation	R2R3-MYB TF	Cotton	Transgenic (RNAi)	Cotton	- Nearly fibre-less seeds.	Crop quality and yield	Walford *et al.* (2011)
MYB109	Trichome formation	R2R3-MYB TF	Cotton	Transgenic (RNAi)	Cotton	- Reduced fibre length.	Crop quality and yield	Pu *et al.* (2008)
MML4	Trichome formation	R2R3-MYB TF	Cotton	Transgenic (VIGS)	Cotton	- Reduced lint fibre initiation.	Crop quality and yield	Wu *et al.* (2018)
GL1	Trichome formation	HD-ZIP I TF	Cucumber	-	-	- Apparent glabrous surfaces with papillae.	Crop quality and yield	Li *et al.* (2015)
Tu/*DULL*	Trichome and cuticle formation	C2H2 zinc-finger TF	Cucumber	Transgenic (OE)<br><br>-	Cucumber<br><br>-	- Promoted warty phenotype in non-warty fruit lines.<br>- Linked to glossy trait and peel cuticle deposition.	Crop quality and yield	Yang *et al.* (2014)<br><br>Zhai *et al.* (2022)

GL3	L1 identity and trichome formation	HD-ZIP IV TF	Cucumber	-	-	- Completely glabrous aerial surfaces.	Crop quality and yield	Pan *et al.* (2015)
TTG1	Trichome formation	WD40-repeat TF	Cucumber	Transgenic (OE/RNAi)	Cucumber	- Increased/reduced fruit trichome density.	Crop quality and yield	Chen *et al.* (2016)
MYB6	Trichome formation	R2R3-MYB TF	Cucumber	Transgenic (OE)	Cucumber	- Reduced fruit trichome and tubercule size and density.	Crop quality and yield	Zhao *et al.* (2020)
TRY	Trichome formation	R3-MYB TF	Cucumber	Transgenic (OE)	Cucumber	- Reduced fruit trichomes.	Crop quality and yield	Yang *et al.* (2018)
CHS1/2	Cuticle formation	Chalcone synthase	Tomato	Transient (VIGS)	Tomato	- Fruit cuticles lacking flavonoids.<br>- Reduced cuticle thickness and viscoelastic behaviour.<br>- Reduced cuticle water loss.	Crop quality and yield	España *et al.* (2014)
MYB12	Cuticle formation	R2R3-MYB TF	Tomato	-	-	- Colourless fruit cuticles.<br>- Reduced fruit cutin.	Crop quality and yield	Adato *et al.* (2009)
				Transgenic (Complement)	Tomato	- Rescued fruit cuticle naringenin chalcone.		
				Transient (VIGS)	Tomato	- Fruit cuticles lacking naringenin chalcone.		Ballester *et al.* (2010)
NOR	Cuticle formation	NAC-domain TF	Tomato	-	-	- Altered wax profiles of fruit cuticles.	Crop quality and yield	Kosma *et al.* (2010)
RIN	Cuticle formation	MADS-box TF	Tomato	-	-	- Altered wax profiles of fruit cuticles.	Crop quality and yield	Kosma *et al.* (2010)
TAGL1	Cuticle formation	MADS-box TF	Tomato	Transgenic (OE/RNAi)	Tomato	- Thicker/thinner fruit cuticles with altered stiffness.	Crop quality and yield	Giménez *et al.* (2015)
FUL1/2	Cuticle formation	MADS-box TF	Tomato	Transgenic (RNAi)	Tomato	- Reduced cuticle lycopenes and repressed cuticle genes.	Crop quality and yield	Bemer *et al.* (2012)
SHN1	Cuticle formation	AP2/ERF-domain TF	Melon	-	-	- Linked to netting density.	Crop quality and yield	Oren *et al.* (2020), Zhang *et al.* (2022)
SHN1	Cuticle formation	AP2/ERF-domain TF	Cucumber	-	-	- Linked to netting density.	Crop quality and yield	Zhang *et al.* (2022)
SHN3	Cuticle formation	AP2/ERF-domain TF	Apple	-	-	- Promote cuticle formation and inhibit russeting.	Crop quality and yield,	Lashbrooke *et al.* (2015b)
				Transgenic (OE)	Arabidopsis	- Increased leaf and stem wax.<br>- Enhanced drought tolerance.	drought tolerance	Y.L. Zhang *et al.* (2019a)
ABCG12	Cuticle formation	ABC transporter	Apple	-	-	- Promote cuticle formation and inhibit russeting.	Crop quality and yield	Falginella *et al.* (2015)
DCR	Cuticle and conical cell formation	BAHD acyltransferase	Tomato	Transgenic (RNAi)	Tomato	- Cracking of fruit skin.<br>- Suberin deposition on fruit surface.	Crop quality and yield	Lashbrooke *et al.* (2016)

SHN3	Cuticle formation	AP2/ERF-domain TF	Tomato	Transgenic (RNAi)	Tomato	- Glossy fruit with reduced cuticle deposition.<br>- Increased fruit water loss and *C. coccodes* susceptibility.	Crop quality and yield, disease resistance	Shi *et al.* (2013)
MIXTA-like	Cuticle, trichome and conical cell formation	R2R3-MYB TF	Tomato	Transgenic (RNAi)<br><br>Transgenic (OE/KO)	Tomato	- Thinner fruit cuticle with flatter epidermal cells.<br>- Increased fruit water loss and *C. coccodes* susceptibility.<br>- Decreased/increased trichome density.<br>- Pronounced/flatter petal epidermal cells.	Crop quality and yield, herbivore defence, disease resistance, pollinator attraction	Lashbrooke *et al.* (2015a)<br><br>Galdon-Armero *et al.* (2020)
CYP86A69	Cuticle formation	CYP450 enzyme	Tomato	-	-	- Reduced fruit cuticle deposition.<br>- Increased fruit water loss and *C. coccodes* susceptibility.	Crop quality and yield, disease resistance	Shi *et al.* (2013)
CD1	Cuticle formation	GDSL Lipase	Tomato	-<br><br>Transgenic (RNAi)	-<br><br>Tomato	- Reduced fruit cutin content.<br>- Increased fruit water loss and *B. cinerea* susceptibility.<br>- Reduced fruit cuticle thickness.<br>- Increased fruit water loss.	Crop quality and yield, disease resistance	Isaacson *et al.* (2009)<br>Yeats *et al.* (2012)<br><br>Girard *et al.* (2012)
CD2	Cuticle and trichome formation	HD-ZIP IV TF	Tomato	-<br><br>-	-<br><br>-	- Reduced fruit cutin content.<br>- Increased fruit susceptibility to *B. cinerea*.<br>- Reduced type-VI leaf trichomes and terpene content.<br>- Reduced leaf cuticle content and stomatal density.<br>- Increased leaf water loss.	Drought tolerance, crop quality and yield, herbivore defence, disease resistance	Isaacson *et al.* (2009)<br><br>Nadakuduti *et al.* (2012)
WAX2	Cuticle formation	-	Cucumber	Transgenic (OE/RNAi)<br><br>Transgenic (OE)	Cucumber<br><br>Arabidopsis	- Increased/decreased leaf and fruit wax.<br>- Decreased/increased fruit water loss.<br>- Increased/decreased fruit fungal growth.<br>- Restored leaf wax content.<br>- Enhanced drought tolerance.	Drought tolerance, crop quality and yield, disease resistance	Wang *et al.* (2015a)
WIN1	Cuticle formation	AP2/ERF-domain TF	Cucumber	-	-	- Glossy fruit surface with thinner cuticle.<br>- Increased wax esters.	Crop quality and yield	J. Zhang *et al.* (2019)

MYB25	Trichome and cuticle formation	R2R3-MYB TF	Peach	-<br>Transgenic (OE)	-<br>Arabidopsis	- Glabrous and shiny nectarine surface.<br>- Increased leaf trichomes and stem waxes.	Crop quality and yield	Vendramin *et al.* (2014)<br>Yang *et al.* (2022)
MYB26	Trichome and cuticle formation	R2R3-MYB TF	Peach	Transgenic (OE)	Arabidopsis	- Increased leaf trichomes and stem waxes.	Crop quality and yield	Yang *et al.* (2022)
SPCH	Stomatal formation	bHLH TF	Tomato	Transgenic (OE)<br>Transgenic (Complement)	Arabidopsis	- Epidermal small-cell clusters.<br>- Rescued stomatal formation.	Drought tolerance	Ortega *et al.* (2019)
MUTE	Stomatal formation	bHLH TF	Tomato	Transgenic (OE)<br>Transgenic (Complement)	Arabidopsis	- Stomata-only epidermis.<br>- Rescued stomatal formation.	Drought tolerance	Ortega *et al.* (2019)
FAMA	Stomatal formation	bHLH TF	Tomato	Transgenic (OE)<br>Transgenic (Complement)	Arabidopsis	- Unpaired guard cells.<br>- Rescued stomatal formation.	Drought tolerance	Ortega *et al.* (2019)
SDD1-like	Stomatal formation	Subtilisin-like Ser protease	Wild Tomato	Transgenic (OE)<br>Transgenic (Complement)	Tomato<br>Arabidopsis	- Reduced stomatal density.<br>- Dehydration avoidance.<br>- Rescued stomatal pattern.<br>- Dehydration avoidance.	Drought tolerance	Morales-Navarro *et al.* (2018)
SPCH1-4	Stomatal formation	bHLH TF	Soybean	Transgenic (RNAi)<br>Transgenic (Complement)	Soybean<br>Arabidopsis	- Lack of stomata.<br>- Rescued stomatal formation.	Drought tolerance	Danzer *et al.* (2015)
FAMA	Stomatal formation	bHLH TF	Rice	GE-NHEJ<br>Transgenic (OE)<br>Transgenic (Complement)	Rice<br>Arabidopsis	- Swollen GCs and SCs<br>- Stomata with one SC.<br>- Unpaired guard cells.<br>- Rescued mature guard cells.	Drought tolerance	Wu *et al.* (2019)<br>Liu *et al.* (2009)
MUTE	Stomatal formation	bHLH TF	Rice	GE-NHEJ<br>Transgenic (OE)	Rice	- Arrested meristemoids.<br>- Epidermal GC-like cells.	Drought tolerance	Wu *et al.* (2019)
MUTE	Stomatal formation	bHLH TF	Maize	GE-NHEJ	Maize	- Absent of SCs.	Drought tolerance	H. Wang *et al.* (2019)
FLP	Stomatal formation	R2R3-MYB TF	Rice	GE-NHEJ	Rice	- Misoriented GMC division resulting in abnormal GCs.	Drought tolerance	Wu *et al.* (2019)
SDD1	Stomatal formation	Subtilisin-like Ser protease	Rice	GE-NHEJ	Rice	- Stomatal clustering with no impact on stomatal density.	Drought tolerance	Yu *et al.* (2020)
SCR1/2	Stomatal formation	GRAS TF	Rice	GE-NHEJ<br>Transgenic (OE)	Rice	- Undivided SCs, arrested meristemoids and reduced stomatal density.<br>- Occasional extra SCs.	Drought tolerance	Wu *et al.* (2019)

SR1/2	Stomatal formation	GRAS TF	Rice	GE-NHEJ<br>Transgenic (OE)	Rice	- Undivided SCs, arrested meristemoids and reduced stomatal density.<br>- Stomata unaffected.	Drought tolerance	Wu *et al.* (2019)
SPCH1/2	Stomatal formation	bHLH TF	Rice	GE-NHEJ<br>Transgenic (OE)	Rice	- Reduced stomatal initiation/density.<br>- Increased stomatal initiation but non-functional.	Drought tolerance	Wu *et al.* (2019)
ICE1/2	Stomatal formation	bHLH TF	Rice	GE-NHEJ<br>Transgenic (OE)	Rice	- Lack of stomata.<br>- Stomata unaffected.	Drought tolerance	Wu *et al.* (2019)
EPF1	Stomatal formation	Signalling peptide	Barley	Transgenic (OE)	Barley	- Arrested meristemoids and GMCs, lower stomatal density.<br>- Improved drought tolerance without yield impact.	Drought tolerance	Hughes *et al.* (2017)
EPF1	Stomatal formation	Signalling peptide	Rice	Transgenic (OE)<br>Transgenic (Complement)	Rice<br>Arabidopsis	- Arrested GMCs, reduced stomatal density.<br>- Improved drought tolerance without yield impact.<br>- Partial rescue of stomatal density and patterning.	Drought tolerance	Caine *et al.* (2019)<br>Lu *et al.* (2019)
EPF1	Stomatal formation	Signalling peptide	Wheat	Transgenic (OE)	Wheat	- Arrested stomatal formation and reduced density.<br>- Improved drought tolerance without yield impact.	Drought tolerance	Dunn *et al.* (2019)
EPF2	Stomatal formation	Signalling peptide	Rice	Transgenic (OE)<br>Transgenic (Complement)	Rice<br>Arabidopsis	- Arrested GMCs and reduced stomatal density.<br>- Rescued stomatal formation and patterning.	Drought tolerance	Lu *et al.* (2019)
EPFL9-1/2	Stomatal formation	Signalling peptide	Rice	Transgenic (RNAi)<br>Transgenic (OE)	Rice<br>Arabidopsis	- Reduced stomatal density.<br>- Increased stomatal density.	Drought tolerance	Lu *et al.* (2019)
EPFL9-1	Stomatal formation	Signalling peptide	Grapevine	GE-NHEJ	Grapevine	- Reduced stomatal density.<br>- Improved WUE.	Drought tolerance	Clemens *et al.* (2022)
SIK1	Stomatal formation	Signalling peptide	Rice	Transgenic (RNAi/OE)	Rice	- Increased/decreased stomatal density.<br>- Impaired/improved drought tolerance.	Drought tolerance	Ouyang *et al.* (2010)
MYB60	Stomatal aperture	R2R3-MYB TF	Grapevine	Transgenic (Complement)	Arabidopsis	- Rescued stomatal opening.	Drought tolerance	Galbiati *et al.* (2011)
TF1L	Stomatal aperture	HD-ZIP IV TF	Rice	Transgenic (OE)	Rice	- Enhanced stomatal closure under drought stimuli.<br>- Improved grain yield.	Drought tolerance,	Bang *et al.* (2019)

							crop quality and yield	
NAC022	Stomatal aperture	NAC TF	Rice	Transgenic (OE)	Rice	- Enhanced stomatal closure and improved drought tolerance.<br>- Reduced grain yield under normal conditions.	Drought tolerance, crop quality and yield	Hong *et al.* (2016)
PIF1/3	Stomatal aperture	bHLH TF	Rice	Transgenic (OE)	Rice	- Enhanced stomatal closure under drought stimuli.<br>- Improved grain yield.	Drought tolerance, crop quality and yield	Gao *et al.*, (2018a), Gao *et al.*, (2018b)
CER1	Cuticle formation	Aldehyde decarbonylase	Apple	Transgenic (OE)	Arabidopsis	- Increased leaf wax.<br>- Enhanced drought tolerance.	Drought tolerance	Qi *et al.* (2019)
KCS2	Cuticle formation	β-ketoacyl-CoA synthase	Apple	Transgenic (OE)	Arabidopsis	- Increased leaf wax.<br>- Enhanced drought tolerance.	Drought tolerance	Lian *et al.* (2021)
LACS1	Cuticle formation	long-chain acyl CoA	Apple	Transgenic (OE)	Arabidopsis	- Increased leaf wax.<br>- Enhanced drought tolerance.	Drought tolerance	Li *et al.* (2022)
ABCG31	Cuticle formation	ABC transporter	Barley	-	-	- Reduced leaf cutin.<br>- Increased leaf water loss.	Drought tolerance	Chen *et al.* (2011)
GDSL1	Cuticle formation	GDSL Lipase	Barley	-	-	- Reduced leaf cutin.<br>- Increased leaf water loss.	Drought tolerance	Li *et al.* (2017)
KCS1	Cuticle formation	β-ketoacyl-CoA synthase	Barley	-	-	- Reduced leaf wax.<br>- Increased leaf water loss.<br>- Reduced *Bgh* germination	Drought tolerance, disease resistance	Li *et al.* (2018)
CER1	Cuticle formation	Aldehyde decarbonylase	Cucumber	Transgenic (OE/RNAi)	Cucumber	- Increased/decreased leaf and fruit wax.<br>- Decreased/increased leaf water loss.	Drought tolerance	Wang *et al.* (2015b)
KCS12l14	Cuticle formation	β-ketoacyl-CoA synthase	Grapevine	Transgenic (OE)	Arabidopsis	- Increased leaf wax.<br>- Enhanced drought tolerance.	Drought tolerance	Liu *et al.* (2023)
ECR	Cuticle formation	Enoyl-CoA reductase	Orange	Transgenic (OE)	Tomato	- Increased leaf wax.<br>- Enhanced drought tolerance.	Drought tolerance	D. Liu *et al.* (2022)
KCS1	Cuticle formation	β-ketoacyl-CoA synthase	Peanut	Transgenic (OE)	Peanut	- Increased leaf wax.<br>- Reduced leaf water loss.	Drought tolerance	Lokesh *et al.* (2019)
WSL1	Cuticle formation	β-ketoacyl-CoA synthase	Rice	Transgenic (KO)	Rice	- Decreased leaf wax.<br>- Enhanced drought sensitivity.	Drought tolerance	Yu *et al.* (2008)
GL1-2	Cuticle formation	-	Rice	Transgenic (OE/KO)	Rice	- Increased/decreased leaf wax.<br>- Enhanced drought sensitivity in mutant (KO).	Drought tolerance	Islam *et al.* (2009)

WDL1	Cuticle formation	GDSL Lipase	Rice	Transgenic (KO)	Rice	- Disorganised leaf cuticle.<br>- Increased leaf water loss.<br>- Stunted plant growth.	Drought tolerance	Park *et al.* (2010)
ABCG31	Cuticle formation	ABC transporter	Rice	Transgenic (KO)	Rice	- Defective leaf cuticle.<br>- Enhanced drought sensitivity.<br>- Stunted plant growth.	Drought tolerance	Chen *et al.* (2011)
GL1-1	Cuticle formation	-	Rice	-	-	- Reduced leaf wax.<br>- Enhanced drought sensitivity.	Drought tolerance	Qin *et al.* (2011)
GL1-6	Cuticle formation	Aldehyde decarbonylase	Rice	Transgenic (RNAi)	Rice	- Decreased leaf wax.<br>- Enhanced drought sensitivity.	Drought tolerance	Zhou *et al.* (2013)
GL1-3	Cuticle formation	-	Rice	Transgenic (OE/RNAi)	Rice	- Increased/decreased leaf wax.<br>- Enhanced drought tolerance, but stunted growth (OE).	Drought tolerance	Zhou *et al.* (2015)
CER1-1	Cuticle formation	Aldehyde decarbonylase	Tomato	Transgenic (RNAi)	Tomato	- Reduced leaf wax.<br>- Increased leaf and fruit water loss.	Drought tolerance, crop quality and yield	Wu *et al.* (2022)
CER1-1A	Cuticle formation	Aldehyde decarbonylase	Wheat	Transgenic (OE)<br><br>Transgenic (OE/Complement)	Rice<br><br>Arabidopsis	- Increased leaf alkanes.<br>- Reduced leaf water loss.<br>- Increased/partially rescued leaf alkanes.<br>- Reduced leaf water loss.	Drought tolerance	Li *et al.* (2019)
WSL5	Cuticle formation	CYP450 enzyme	Rice	Transgenic (OE)/ GE-NHEJ<br>-	Rice<br><br>-	- Altered leaf wax composition.<br><br>- Enhanced drought tolerance.	Drought tolerance	Zhang *et al.* (2020)
DMC	Cuticle formation	CYP450 enzyme	Wheat	-	-	- Linked to glaucous surface.	Drought tolerance	Hen-Avivi *et al.* (2016)
DMH	Cuticle formation	Hydrolase	Wheat	Transgenic (VIGS)	Wheat	- Decreased leaf wax.	Drought tolerance	Hen-Avivi *et al.* (2016)
DMP	Cuticle formation	Polyketide synthase	Wheat	Transgenic (VIGS)	Wheat	- Decreased leaf wax.	Drought tolerance	Hen-Avivi *et al.* (2016)
SHN1/2	Cuticle formation	AP2/ERF-domain TF	Papaya	-	-	- Increased expression promotes wax accumulation under water deficiency.	Drought tolerance	Girón-Ramírez *et al.* (2021)
WR1	Cuticle formation	AP2/ERF-domain TF	Rice	Transgenic (OE/RNAi)	Rice	- Increased/reduced leaf wax.<br>- Enhanced drought tolerance/sensitivity.	Drought tolerance	Wang *et al.* (2012)
WR2	Cuticle formation	AP2/ERF-domain TF	Rice	Transgenic (OE)	Rice	- Increased leaf cutin and wax.<br>- Enhanced drought tolerance.<br>- Partial male sterility.	Drought tolerance	Zhou *et al.* (2014)

WIN-like1	Cuticle formation	AP2/ERF-domain TF	Sorghum	Transgenic (OE)	Arabidopsis	- Increased leaf cutin and wax.<br>- Enhanced drought tolerance.	Drought tolerance	Bao *et al.* (2017)
SHN1	Cuticle formation	AP2/ERF-domain TF	Tomato	Transgenic (OE)	Tomato	- Increased leaf wax.<br>- Enhanced drought tolerance.<br>- Mild growth retardation.	Drought tolerance	Al-Abdallat *et al.* (2014)
SHN1	Stomatal and cuticle formation	AP2/ERF-domain TF	Wheat	Transgenic (OE)	Tobacco	- Upregulated wax genes.<br>- Enhanced drought tolerance.<br>- Reduced stomatal density.	Drought tolerance	Djemal and Khoudi (2016)
				Transgenic (OE)	Wheat	- Increased leaf wax.<br>- Enhanced drought tolerance without yield impact.<br>- Reduced stomatal density.		Bi *et al.* (2018)
SHN1/9	Cuticle formation	AP2/ERF-domain TF	Soybean	Transgenic (OE)	Arabidopsis	- Increased leaf wax and cutin.<br>- Loosely assembled cuticle.<br>- Increased leaf water loss.	Drought tolerance	Xu *et al.* (2016)
MX1	Cuticle and trichome formation	R2R3-MYB TF	Tomato	Transgenic (OE/RNAi)	Tomato	- Increased/reduced fruit cuticle thickness.<br>- Increased/reduced trichome density.<br>- Reduced/increased leaf water loss.	Drought tolerance, herbivore defence	Ewas *et al.* (2016)
MYB60	Cuticle formation	R2R3-MYB TF	Rice	GE-NHEJ	Rice	- Reduced leaf wax.<br>- Enhanced drought sensitivity.	Drought tolerance	Jian *et al.* (2022)
MYB94	Cuticle formation	R2R3-MYB TF	Maize	-	-	- Fused young leaves due to irregular cuticle deposition.<br>- Increased plant water loss.<br>- Stunted plant growth.	Drought tolerance	La Rocca *et al.* (2015), Castorina *et al.* (2020)
CHR4	Cuticle formation	CHD3 protein	Rice	-	-	- Increased leaf wax.<br>- Enhanced drought tolerance.	Drought tolerance	Guo *et al.* (2019)
DHS	Cuticle formation	RING-type E3 ligase	Rice	Transgenic (OE)	Rice	- Decreased leaf wax.<br>- Enhanced drought sensitivity.<br>- Stunted plant growth.	Drought tolerance	Wang *et al.* (2018)
ROC4	Cuticle formation	HD-ZIP IV TF	Rice	Transgenic (OE)	Rice	- Increased leaf wax.<br>- Enhanced drought tolerance.	Drought tolerance	Wang *et al.* (2018)
Wo	L1 identity, cuticle and trichome formation	HD-ZIP IV TF	Tomato	-	-	- Increased type-IV juvenile leaf trichomes.	Herbivore defence	Vendemiatti *et al.* (2017)
H	Trichome formation	C2H2 zinc-finger TF	Tomato	Transgenic (OE/RNAi)	Tomato	- Increased/eliminated type-I trichomes.	Herbivore defence	Chang *et al.* (2018)
				Transgenic (OE)	Tobacco	- Increased trichome density.		
CycB2	Trichome formation	B-type cyclin	Tomato	Transgenic (OE)	Tomato	- Reduced leaf trichome density and terpene content.	Herbivore defence	Gao *et al.* (2017)

						- Increased susceptibility to *P. litura* herbivory.		
ZFP8L	Trichome formation	C2H2 zinc-finger TF	Tomato	Transgenic (OE)	Tomato	- Increased leaf trichome density and length.<br>- Dwarfed plants.	Herbivore defence	Zheng *et al.* (2022)
ZFP6	Trichome formation	C2H2 zinc-finger TF	Tomato	Transgenic (OE)	Tomato	- Increased leaf trichome density and length.<br>- Dwarfed plants.	Herbivore defence	Zheng *et al.* (2022)
MYB75	Trichome formation	R2R3-MYB TF	Tomato	Transgenic (OE/RNAi)	Tomato	- Lower/higher leaf trichome density and terpene content.<br>- Increased/reduced susceptibility to spider mite infestation.	Herbivore defence	Gong *et al.* (2021)
MYC1	Trichome formation	bHLH TF	Tomato	Transgenic (RNAi)	Tomato	- Reduced type-VI leaf trichomes and terpene content.	Herbivore defence	Xu *et al.* (2018)
SRA1	Trichome formation	SCAR/WAVE complex subunit	Tomato	Transgenic (Complement)	Tomato	- Restored trichome formation, terpene synthesis and *M. sexta* defence.	Herbivore defence	Kang *et al.* (2016)
ARPC2A	Trichome formation	ARP2/3 complex subunit	Tomato	-	-	- Bent and swollen trichomes.	Herbivore defence	Jeong *et al.* (2017)
Hl-2	Trichome formation	SCAR/WAVE complex subunit	Tomato	GE-NHEJ<br>Transgenic (Complement)	Tomato	- Distorted trichomes.<br>- Restored normal trichome development.	Herbivore defence	Q. Xie *et al.* (2020)
HDZIV8	Trichome formation	HD-ZIP IV TF	Tomato	Transgenic (RNAi)	Tomato	- Distorted trichomes.<br>- Reduced trichome density.	Herbivore defence	Q. Xie *et al.* (2020)
CHR729	Cuticle formation	CHD3 protein	Wheat	Transgenic (BSMV)	Wheat	- Decreased leaf wax and *Bgt* germination rates.	Disease resistance	X. Wang *et al.* (2019)
KPAB1	Cuticle formation	bHLH TF	Wheat	Transgenic (BSMV)	Wheat	- Decreased leaf wax and *Bgt* germination rates.	Disease resistance	X. Wang *et al.* (2019)
KCS6	Cuticle formation	β-ketoacyl-CoA synthase	Wheat	Transgenic (BSMV)	Wheat	- Decreased leaf wax and *Bgt* germination rates.	Disease resistance	X. Wang *et al.* (2019)
ECR	Cuticle formation	Enoyl-CoA Reductase	Wheat	Transgenic (BSMV)	Wheat	- Reduced leaf wax.<br>- Decreased *Bgt* germination.	Disease resistance	Kong *et al.* (2020)
KCS6	Cuticle formation	β-ketoacyl-CoA synthase	Barley	Transgenic (Complement)	Barley	- Restored leaf wax and *Bgh* germination rates.	Disease resistance	Weidenbach *et al.* (2014)
WIN1	Cuticle formation	AP2/ERF-domain TF	Barley	Transgenic (VIGS)	Barley	- Downregulated cutin genes.<br>- Enhanced susceptibility to Fusarium head blight.	Disease resistance	Kumar *et al.* (2016)
MYB30	Cuticle formation	R2R3-MYB TF	Apple	Transgenic (OE)	Apple	- Enhanced *B. dothidea* resistance.	Disease resistance	Y.L. Zhang *et al.* (2019b)

				Transgenic (OE)	Arabidopsis	- Enhanced *Pst* resistance.<br>- Increased leaf wax.		
GPAT6	Cuticle formation	Glycerol-3-phosphate acyltransferase	Tomato	-	-	- Increased leaf cuticle thickness.<br>- Enhanced *Phytophthora* susceptibility and *B. cinerea* resistance.	Disease resistance	Fawke *et al.* (2019)
OSC4/5	Cuticle formation	Oxidosqualene cyclase	Apple	Transient (OE)	*N. bent.*	- Increased triterpene content in leaves.	Disease resistance	Andre *et al.* (2016)
OSC2	Cuticle formation	Oxidosqualene cyclase	Sweet wormwood	Transient (OE)	*N. bent.*	- Increased amyrin production in leaves.	Disease resistance	Moses *et al.* (2015)
TTS1/2	Cuticle formation	Oxidosqualene cyclase	Tomato	Transgenic (OE)	Tomato	- Increased fruit cuticle amyrin content.	Disease resistance	Z. Wang *et al.* (2011)
CYP716A175	Cuticle formation	CYP450 enzyme	Apple	Transient (OE)	*N. bent.*	- Co-expression with OSC4/5 leads to leaf triterpene C-28 diversification.	Disease resistance	Andre *et al.* (2016)
CYP716A14v2	Cuticle formation	CYP450 enzyme	Sweet wormwood	Transient (OE)	*N. bent.*	- Co-expression with OSC2 leads to leaf triterpene C-3 diversification.	Disease resistance	Moses *et al.* (2015)
MYB5b	Cuticle formation	R2R3-MYB TF	Grapevine	Transgenic (OE)	Tomato	- Reduced fruit cuticle amyrin content.<br>- Pleiotropic traits in vegetative and floral organ growth.	Disease resistance	Mahjoub *et al.* (2009)
MYB9A1/2	Cuticle and conical cell formation	R2R3-MYB TF	Orchid	Transient (OE/SRDX)<br>Transgenic (OE)	Orchid<br>*N. bent.*	- Increased petal wax (OE).<br>- Flattened adaxial petal epidermal cells (SRDX).<br>- Shiny abaxial leaf surfaces with altered wax profiles.	Pollinator attraction	Lu *et al.* (2022)
MYB1	Conical cell formation	R2R3-MYB TF	Petunia	Transgenic (OE)<br>-	Tobacco<br>-	- Extended the conical cells of petals.<br>- Paler petals with altered architecture.	Pollinator attraction	Baumann *et al.* (2007)
MIXTA	Conical cell formation	R2R3-MYB TF	Snapdragon	-	-	- Paler petals with altered architecture.	Pollinator attraction	Baumann *et al.* (2007)
MYBML2	Conical cell formation	R2R3-MYB TF	Snapdragon	Transgenic (OE)	Tobacco	- Extended the conical cells of petals.	Pollinator attraction	Baumann *et al.* (2007)
ERF1	Cuticle formation	AP2/ERF-domain TF	Orchid	Transgenic (VIGS)<br>Transgenic (OE)	Orchid<br>Arabidopsis	- Fewer lip nanoridges and repressed cuticle genes.<br>- Shiny leaves with increased wax deposition.	Pollinator attraction	Lai *et al.* (2020)

APV1	Cuticle formation	CYP450 enzyme	Maize	-	-	- Deformed anthers with reduced cutin.<br>- Male sterility.	Crop fertility	Somaratne *et al.* (2017)
Ms33	Cuticle formation	Glycerol-3-phosphate acyltransferase	Maize	GE-NHEJ<br><br><br>Transgenic (Complement)	Maize	- Deformed anthers with impaired cuticle.<br>- Male sterility.<br>- Rescued anther development and male fertility.	Crop fertility	Xie *et al.* (2018)
Ms30	Cuticle formation	GDSL Lipase	Maize	GE-NHEJ<br><br><br>Transgenic (Complement)	Maize	- Deformed anthers with impaired cuticle.<br>- Male sterility.<br>- Rescued anther development and male fertility.	Crop fertility	An *et al.* (2019)
FAR1	Cuticle formation	Fatty acyl reductase	Maize	GE-NHEJ	Maize	- Deformed anthers with reduced cutin.<br>- Male sterility.	Crop fertility	Jiang *et al*. (2021)
Ms25	Cuticle formation	Fatty acyl reductase	Maize	GE-NHEJ	Maize	- Deformed anthers with impaired cuticle.<br>- Male sterility.	Crop fertility	S. Zhang *et al.* (2021)
Wda1	Cuticle formation	Aldehyde decarbonylase	Rice	Transgenic (KO)	Rice	- Deformed anthers with impaired cuticle.<br>- Male sterility.	Crop fertility	Jung *et al.* (2006)
GPAT3	Cuticle formation	Glycerol-3-phosphate acyltransferase	Rice	Transgenic (KO)<br><br><br>Transgenic (Complement)	Rice	- Deformed anthers with impaired cuticle.<br>- Male sterility.<br>- Rescued anther development and male fertility.	Crop fertility	Men *et al.* (2017)
HTH1	Cuticle formation	GMC oxidoreductase	Rice	Transgenic (RNAi)	Rice	- Deformed anthers with impaired cuticle.<br>- Reduced male fertility.	Crop fertility	Xu *et al.* (2017)
RMS2	Cuticle formation	GDSL Lipase	Rice	GE-NHEJ<br><br><br>Transgenic (Complement)	Rice	- Deformed anthers with impaired cuticle.<br>- Male sterility.<br>- Rescued anther development and male fertility.	Crop fertility	J. Zhao *et al.* (2020)
ABCG26	Cuticle formation	ABC transporter	Rice	-<br><br><br>Transgenic (Complement)	-	- Deformed anthers with reduced cutin.<br>- Male sterility.<br>- Rescued anther development and male fertility.	Crop fertility	Zhao *et al.* (2015)

ABCG26	Cuticle formation	ABC transporter	Maize	GE-NHEJ	Maize	- Deformed anthers with reduced cutin.<br>- Male sterility.	Crop fertility	Jiang *et al*. (2021)

*Genes are listed according to the order in which they are discussed in the text.

References

Adato A, Mandel T, Mintz-Oron S, *et al.* 2009. Fruit-surface flavonoid accumulation in tomato is controlled by a SlMYB12-regulated transcriptional network. PLoS Genetics **5**, e1000777.

Al-Abdallat AM, Al-Debei HS, Ayad JY, Hasan S. 2014. Over-expression of SlSHN1 gene improves drought tolerance by increasing cuticular wax accumulation in tomato. International Journal of Molecular Sciences **15**, 19499-19515.

An X, Dong Z, Tian Y, *et al.* 2019. ZmMs30 encoding a novel GDSL lipase is essential for male fertility and valuable for hybrid breeding in maize. Molecular Plant **12**, 343-359.

Andre CM, Legay S, Deleruelle A, *et al*. 2016. Multifunctional oxidosqualene cyclases and cytochrome P450 involved in the biosynthesis of apple fruit triterpenic acids. New Phytologist **211**, 1279-1294.

Ballester AR, Molthoff J, de Vos R, *et al.* 2010. Biochemical and molecular analysis of pink tomatoes: deregulated expression of the gene encoding transcription factor SlMYB12 leads to pink tomato fruit color. Plant Physiology **152**, 71-84.

Bang SW, Lee DK, Jung H, Chung PJ, Kim YS, Choi YD, Suh JW, Kim JK. 2019. Overexpression of OsTF1L, a rice HD-Zip transcription factor, promotes lignin biosynthesis and stomatal closure that improves drought tolerance. Plant Biotechnology Journal **17**, 118-131.

Bao SG, Shi JX, Luo F, Ding B, Hao JY, Xie XD, Sun SJ. 2017. Overexpression of Sorghum WINL1 gene confers drought tolerance in Arabidopsis thaliana through the regulation of cuticular biosynthesis. Plant Cell, Tissue and Organ Culture (PCTOC) **128**, 347-356.

Baumann K, Perez-Rodriguez M, Bradley D, Venail J, Bailey P, Jin H, Koes R, Roberts K, Martin C. 2007. Control of cell and petal morphogenesis by R2R3 MYB transcription factors. Development **134**, 1691-1701.

Becraft PW, Li K, Dey N, Asuncion-Crabb Y. 2002. The maize dek1 gene functions in embryonic pattern formation and cell fate specification. Development **129**, 5217-5225.

Becraft PW, Stinard PS, McCarty DR. 1996. CRINKLY4: a TNFR-like receptor kinase involved in maize epidermal differentiation. Science **273**, 1406-1409.

Bemer M, Karlova R, Ballester AR, Tikunov YM, Bovy AG, Wolters-Arts M, Rossetto PDB, Angenent GC, de Maagd RA. 2012. The tomato FRUITFULL homologs TDR4/FUL1 and MBP7/FUL2 regulate ethylene-independent aspects of fruit ripening. The Plant Cell **24**, 4437-4451.

Bi H, Shi J, Kovalchuk N, *et al.* 2018. Overexpression of the TaSHN1 transcription factor in bread wheat leads to leaf surface modifications, improved drought tolerance, and no yield penalty under controlled growth conditions. Plant, Cell & Environment **41**, 2549-2566.

Caine RS, Yin X, Sloan J, *et al.* 2019. Rice with reduced stomatal density conserves water and has improved drought tolerance under future climate conditions. New Phytologist **221**, 371-384.

Castorina G, Domergue F, Chiara M, Zilio M, Persico M, Ricciardi V, Horner DS, Consonni G. 2020. Drought-responsive ZmFDL1/MYB94 regulates cuticle biosynthesis and cuticle-dependent leaf permeability. Plant Physiology **184**, 266-282.

Chang J, Yu T, Yang Q, *et al.* 2018. Hair, encoding a single C2H2 zinc-finger protein, regulates multicellular trichome formation in tomato. The Plant Journal **96**, 90-102.

Chen C, Yin S, Liu X, *et al.* 2016. The WD-repeat protein CsTTG1 regulates fruit wart formation through interaction with the homeodomain-leucine zipper I protein Mict. Plant Physiology **171**, 1156-1168.

Chen G, Komatsuda T, Ma JF, *et al.* 2011. An ATP-binding cassette subfamily G full transporter is essential for the retention of leaf water in both wild barley and rice. Proceedings of the National Academy of Sciences **108**, 12354-12359.

Chun Y, Fang J, Zafar SA, Shang J, Zhao J, Yuan S, Li X. 2020. MINI SEED 2 (MIS2) encodes a receptor-like kinase that controls grain size and shape in rice. Rice **13**, 1-17.

Clemens M, Faralli M, Lagreze J, Bontempo L, Piazza S, Varotto C, Malnoy M, Oechel W, Rizzoli A, Dalla Costa L. 2022. VvEPFL9-1 Knock-out via CRISPR/Cas9 reduces Stomatal density in grapevine. Frontiers in Plant Science **13**, 878001.

Danzer J, Mellott E, Bui AQ, *et al.* 2015. Down-regulating the expression of 53 soybean transcription factor genes uncovers a role for SPEECHLESS in initiating stomatal cell lineages during embryo development. Plant Physiology **168**, 1025-1035.

Deng F, Tu L, Tan J, Li Y, Nie Y, Zhang X. 2012. GbPDF1 is involved in cotton fiber initiation via the core cis-element HDZIP2ATATHB2. Plant Physiology **158**, 890-904.

Djemal R, Khoudi H. 2016. TdSHN1, a WIN1/SHN1-type transcription factor, imparts multiple abiotic stress tolerance in transgenic tobacco. Environmental and Experimental Botany **131**, 89-100.

Dunn J, Hunt L, Afsharinafar M, Meselmani MA, Mitchell A, Howells R, Wallington E, Fleming AJ, Gray JE. 2019. Reduced stomatal density in bread wheat leads to increased water-use efficiency. Journal of Experimental Botany **70**, 4737-4748.

España L, Heredia-Guerrero JA, Reina-Pinto JJ, Fernández-Muñoz R, Heredia A, Domínguez E. 2014. Transient silencing of CHALCONE SYNTHASE during fruit ripening modifies tomato epidermal cells and cuticle properties. Plant Physiology **166**, 1371-1386.

Ewas M, Gao Y, Wang S, *et al.* 2016. Manipulation of SlMXl for enhanced carotenoids accumulation and drought resistance in tomato. Science Bulletin **61**, 1413-1418.

Falginella L, Cipriani G, Monte C, Gregori R, Testolin R, Velasco R, Troggio M, Tartarini S. 2015. A major QTL controlling apple skin russeting maps on the linkage group 12 of 'Renetta Grigia di Torriana'. BMC Plant Biology **15**, 1-13.

Fawke S, Torode TA, Gogleva A, Fich EA, Sørensen I, Yunusov T, Rose JK, Schornack S. 2019. Glycerol-3-phosphate acyltransferase 6 controls filamentous pathogen interactions and cell wall properties of the tomato and Nicotiana benthamiana leaf epidermis. New Phytologist **223**, 1547-1559.

Galbiati M, Matus JT, Francia P, Rusconi F, Cañón P, Medina C, Conti L, Cominelli E, Tonelli C, Arce-Johnson P. 2011. The grapevine guard cell-related VvMYB60 transcription factor is involved in the regulation of stomatal activity and is differentially expressed in response to ABA and osmotic stress. BMC Plant Biology **11**, 1-15.

Galdon-Armero J, Arce-Rodriguez L, Downie M, Li J, Martin C. 2020. A scanning electron micrograph-based resource for identification of loci involved in epidermal development in tomato: elucidation of a new function for the Mixta-like transcription factor in leaves. The Plant Cell **32**, 1414-1433.

Gao S, Gao Y, Xiong C, Yu G, Chang J, Yang Q, Yang C, Ye Z. 2017. The tomato B-type cyclin gene, SlCycB2, plays key roles in reproductive organ development, trichome initiation, terpenoids biosynthesis and Prodenia litura defense. Plant Science **262**, 103-114.

Gao Y, Wu M, Zhang M, Jiang W, Liang E, Zhang D, Zhang C, Xiao N, Chen J. 2018a. Roles of a maize phytochrome-interacting factors protein ZmPIF3 in regulation of drought stress responses by controlling stomatal closure in transgenic rice without yield penalty. Plant Molecular Biology **97**, 311-323.

Gao Y, Wu M, Zhang M, *et al.* 2018b. A maize phytochrome-interacting factors protein ZmPIF1 enhances drought tolerance by inducing stomatal closure and improves grain yield in Oryza sativa. Plant Biotechnology Journal **16**, 1375-1387.

Giménez E, Dominguez E, Pineda B, Heredia A, Moreno V, Lozano R, Angosto T. 2015. Transcriptional activity of the MADS box ARLEQUIN/TOMATO AGAMOUS-LIKE1 gene is required for cuticle development of tomato fruit. Plant Physiology **168**, 1036-1048.

Girard AL, Mounet F, Lemaire-Chamley M, *et al*. 2012. Tomato GDSL1 is required for cutin deposition in the fruit cuticle. The Plant Cell **24**, 3119-3134.

Girón-Ramírez A, Peña-Rodríguez LM, Escalante-Erosa F, Fuentes G, Santamaría JM. 2021. Identification of the SHINE clade of AP2/ERF domain transcription factors genes in Carica papaya; Their gene expression and their possible role in wax accumulation and water deficit stress tolerance in a wild and a commercial papaya genotypes. Environmental and Experimental Botany **183**, 104341.

Gong Z, Luo Y, Zhang W, *et al.* 2021. A SlMYB75-centred transcriptional cascade regulates trichome formation and sesquiterpene accumulation in tomato. Journal of Experimental Botany **72**, 3806-3820.

Guan XY, Li QJ, Shan CM, Wang S, Mao YB, Wang LJ, Chen XY. 2008. The HD-Zip IV gene GaHOX1 from cotton is a functional homologue of the Arabidopsis GLABRA2. Physiologia Plantarum **134**, 174-182.

Guo T, Wang D, Fang J, Zhao J, Yuan S, Xiao L, Li X. 2019. Mutations in the rice OsCHR4 gene, encoding a CHD3 family chromatin remodeler, induce narrow and rolled leaves with increased cuticular wax. International Journal of Molecular Sciences **20**, 2567.

Hen-Avivi S, Savin O, Racovita RC, *et al.* 2016. A metabolic gene cluster in the wheat W1 and the barley Cer-cqu loci determines β-diketone biosynthesis and glaucousness. The Plant Cell **28**, 1440-1460.

Hibara KI, Obara M, Hayashida E, Abe M, Ishimaru T, Satoh H, Itoh JI, Nagato Y. 2009. The ADAXIALIZED LEAF1 gene functions in leaf and embryonic pattern formation in rice. Developmental Biology **334**, 345-354.

Hong Y, Zhang H, Huang L, Li D, Song F. 2016. Overexpression of a stress-responsive NAC transcription factor gene ONAC022 improves drought and salt tolerance in rice. Frontiers in Plant Science **7**, 4.

Hughes J, Hepworth C, Dutton C, Dunn JA, Hunt L, Stephens J, Waugh R, Cameron DD, Gray JE. 2017. Reducing stomatal density in barley improves drought tolerance without impacting on yield. Plant Physiology **174**, 776-787.

Humphries JA, Walker AR, Timmis JN, Orford SJ. 2005. Two WD-repeat genes from cotton are functional homologues of the Arabidopsis thaliana TRANSPARENT TESTA GLABRA1 (TTG1) gene. Plant Molecular Biology **57**, 67-81.

Isaacson T, Kosma DK, Matas AJ, *et al.* 2009. Cutin deficiency in the tomato fruit cuticle consistently affects resistance to microbial infection and biomechanical properties, but not transpirational water loss. The Plant Journal **60**, 363-377.

Islam MA, Du H, Ning J, Ye H, Xiong L. 2009. Characterization of Glossy1-homologous genes in rice involved in leaf wax accumulation and drought resistance. Plant Molecular Biology **70**, 443-456.

Jeong NR, Kim H, Hwang IT, Howe GA, Kang JH. 2017. Genetic analysis of the tomato inquieta mutant links the ARP2/3 complex to trichome development. Journal of Plant Biology **60**, 582-592.

Jian L, Kang K, Choi Y, Suh MC, Paek NC. 2022. Mutation of OsMYB60 reduces rice resilience to drought stress by attenuating cuticular wax biosynthesis. The Plant Journal **112**, 339-351.

Jiang Y, Li Z, Liu X, *et al*. 2021. ZmFAR1 and ZmABCG26 regulated by microRNA are essential for lipid metabolism in maize anther. International Journal of Molecular Sciences **22**, 7916.

Jin P, Guo T, Becraft PW. 2000. The maize CR4 receptor-like kinase mediates a growth factor-like differentiation response. Genesis **27**, 104-116.

Jung KH, Han MJ, Lee DY, *et al.* 2006. Wax-deficient anther1 is involved in cuticle and wax production in rice anther walls and is required for pollen development. The Plant Cell **18**, 3015-3032.

Kang JH, Campos ML, Zemelis-Durfee S, Al-Haddad JM, Jones AD, Telewski FW, Brandizzi F, Howe GA. 2016. Molecular cloning of the tomato Hairless gene implicates actin dynamics in trichome-mediated defense and mechanical properties of stem tissue. Journal of Experimental Botany **67**, 5313-5324.

Kong L, Zhi P, Liu J, Li H, Zhang X, Xu J, Zhou J, Wang X, Chang C. 2020. Epigenetic activation of Enoyl-CoA Reductase by an acetyltransferase complex triggers wheat wax biosynthesis. Plant Physiology **183**, 1250-1267.

Kosma DK, Parsons EP, Isaacson T, Lü S, Rose JK, Jenks MA. 2010. Fruit cuticle lipid composition during development in tomato ripening mutants. Physiologia Plantarum **139**, 107-117.

Kumar A, Yogendra KN, Karre S, Kushalappa AC, Dion Y, Choo TM. 2016. WAX INDUCER1 (HvWIN1) transcription factor regulates free fatty acid biosynthetic genes to reinforce cuticle to resist Fusarium head blight in barley spikelets. Journal of Experimental Botany **67**, 4127-4139.

Lai PH, Huang LM, Pan ZJ, Jane WN, Chung MC, Chen WH, Chen HH. 2020. PeERF1, a SHINE-like transcription factor, is involved in nanoridge development on lip epidermis of phalaenopsis flowers. Frontiers in Plant Science **10**, 1709.

La Rocca N, Manzotti PS, Cavaiuolo M, *et al.* 2015. The maize fused leaves1 (fdl1) gene controls organ separation in the embryo and seedling shoot and promotes coleoptile opening. Journal of Experimental Botany **66**, 5753-5767.

Lashbrooke J, Adato A, Lotan O, *et al.* 2015a. The tomato MIXTA-like transcription factor coordinates fruit epidermis conical cell development and cuticular lipid biosynthesis and assembly. Plant Physiology **169**, 2553-2571.

Lashbrooke J, Aharoni A, Costa F. 2015b. Genome investigation suggests MdSHN3, an APETALA2-domain transcription factor gene, to be a positive regulator of apple fruit cuticle formation and an inhibitor of russet development. Journal of Experimental Botany **66**, 6579-6589.

Lashbrooke J, Cohen H, Levy-Samocha D, *et al.* 2016. MYB107 and MYB9 homologs regulate suberin deposition in angiosperms. The Plant Cell **28**, 2097-2116.

Li C, Chen G, Mishina K, *et al.* 2017. A GDSL-motif esterase/acyltransferase/lipase is responsible for leaf water retention in barley. Plant Direct **1**, e00025.

Li C, Haslam TM, Krüger A, *et al.* 2018. The β-ketoacyl-CoA synthase HvKCS1, encoded by Cer-zh, plays a key role in synthesis of barley leaf wax and germination of barley powdery mildew. Plant and Cell Physiology **59**, 811-827.

Li JJ, Zhang CL, Zhang YL, Gao HN, Wang HB, Jiang H, Li YY. 2022. An apple long-chain acyl-CoA synthase, MdLACS1, enhances biotic and abiotic stress resistance in plants. Plant Physiology and Biochemistry **189**, 115-125.

Li Q, Cao C, Zhang C, Zheng S, Wang Z, Wang L, Ren Z. 2015. The identification of Cucumis sativus Glabrous 1 (CsGL1) required for the formation of trichomes uncovers a novel function for the homeodomain-leucine zipper I gene. Journal of Experimental Botany **66**, 2515-2526.

Li T, Sun Y, Liu T, *et al.* 2019. TaCER1-1A is involved in cuticular wax alkane biosynthesis in hexaploid wheat and responds to plant abiotic stresses. Plant, Cell & Environment **42**, 3077-3091.

Lian XY, Gao HN, Jiang H, Liu C, Li YY. 2021. MdKCS2 increased plant drought resistance by regulating wax biosynthesis. Plant Cell Reports **40**, 2357-2368.

Liu B, Sun Y, Li X, Guo D, Zhao L, Ma C, Wang L, Wang S. 2023. β-ketoacyl-CoA synthase improves the drought tolerance of root restricted grown grapevines by regulating the cuticular wax biosynthesis. Scientia Horticulturae **307**, 111494.

Liu B, Zhu Y, Zhang T. 2015. The R3-MYB gene GhCPC negatively regulates cotton fiber elongation. PloS ONE **10**, e0116272.

Liu D, Guo W, Guo X, Yang L, Hu W, Kuang L, Huang Y, Xie J, Liu Y. 2022. Ectopic overexpression of CsECR from navel orange increases cuticular wax accumulation in tomato and enhances its tolerance to drought stress. Frontiers in Plant Science **13**, 924552.

Liu N, Chen J, Wang T, Li Q, Cui P, Jia C, Hong Y. 2019. Overexpression of WAX INDUCER1/SHINE1 gene enhances wax accumulation under osmotic stress and oil synthesis in Brassica napus. International Journal of Molecular Sciences **20**, 4435.

Liu T, Ohashi-Ito K, Bergmann DC. 2009. Orthologs of Arabidopsis thaliana stomatal bHLH genes and regulation of stomatal development in grasses. Development **136**, 2265–2276.

Lokesh U, Venkatesh B, Kiranmai K, Nareshkumar A, Amarnathareddy V, Rao GL, Anthony Johnson AM, Pandurangaiah M, Sudhakar C. 2019. Overexpression of ß-Ketoacyl Co-A Synthase1 gene improves tolerance of drought susceptible groundnut (Arachis hypogaea L.) cultivar K-6 by increased leaf epicuticular wax accumulation. Frontiers in Plant Science **9**, 1869.

Lu HC, Lam SH, Zhang D, Hsiao YY, Li BJ, Niu SC, Li CY, Lan S, Tsai WC, Liu ZJ. 2022. R2R3-MYB genes coordinate conical cell development and cuticular wax biosynthesis in Phalaenopsis aphrodite. Plant Physiology **188**, 318-331.

Lu J, He J, Zhou X, Zhong J, Li J, Liang YK. 2019. Homologous genes of epidermal patterning factor regulate stomatal development in rice. Journal of Plant Physiology **234**, 18-27.

Machado A, Wu Y, Yang Y, Llewellyn DJ, Dennis ES. 2009. The MYB transcription factor GhMYB25 regulates early fibre and trichome development. The Plant Journal **59**, 52-62.

Mahjoub A, Hernould M, Joubès J, Decendit A, Mars M, Barrieu F, Hamdi S, Delrot S. 2009. Overexpression of a grapevine R2R3-MYB factor in tomato affects vegetative development, flower morphology and flavonoid and terpenoid metabolism. Plant Physiology and Biochemistry **47**, 551-561.

Men X, Shi J, Liang W, Zhang Q, Lian G, Quan S, Zhu L, Luo Z, Chen M, Zhang D. 2017. Glycerol-3-Phosphate Acyltransferase 3 (OsGPAT3) is required for anther development and male fertility in rice. Journal of Experimental Botany **68**, 513-526.

Morales-Navarro S, Pérez-Díaz R, Ortega A, De Marcos A, Mena M, Fenoll C, González-Villanueva E, Ruiz-Lara S. 2018. Overexpression of a SDD1-like gene from wild tomato decreases stomatal density and enhances dehydration avoidance in Arabidopsis and cultivated tomato. Frontiers in Plant Science **9**, 940.

Moses T, Pollier J, Shen Q, *et al.* 2015. OSC2 and CYP716A14v2 catalyze the biosynthesis of triterpenoids for the cuticle of aerial organs of Artemisia annua. The Plant Cell **27**, 286-301.

Nadakuduti SS, Pollard M, Kosma DK, Allen Jr C, Ohlrogge JB, Barry CS. 2012. Pleiotropic phenotypes of the sticky peel mutant provide new insight into the role of CUTIN DEFICIENT2 in epidermal cell function in tomato. Plant Physiology **159**, 945-960.

Oren E, Tzuri G, Dafna A, *et al.* 2020. High-density NGS-based map construction and genetic dissection of fruit shape and rind netting in Cucumis melo. Theoretical and Applied Genetics **133**, 1927-1945.

Ortega A, De Marcos A, Illescas-Miranda J, Mena M, Fenoll C. 2019. The tomato genome encodes SPCH, MUTE, and FAMA candidates that can replace the endogenous functions of their Arabidopsis orthologs. Frontiers in Plant Science **10**, 1300.

Ouyang SQ, Liu YF, Liu P, Lei G, He SJ, Ma B, Zhang WK, Zhang JS, Chen SY. 2010. Receptor-like kinase OsSIK1 improves drought and salt stress tolerance in rice (Oryza sativa) plants. The Plant Journal **62**, 316-329.

Pan Y, Bo K, Cheng Z, Weng Y. 2015. The loss-of-function GLABROUS 3 mutation in cucumber is due to LTR-retrotransposon insertion in a class IV HD-ZIP transcription factor gene CsGL3 that is epistatic over CsGL1. BMC Plant Biology **15**, 1-15.

Park JJ, Jin P, Yoon J, Yang JI, Jeong HJ, Ranathunge K, Schreiber L, Franke R, Lee IJ, An G. 2010. Mutation in Wilted Dwarf and Lethal 1 (WDL1) causes abnormal cuticle formation and rapid water loss in rice. Plant Molecular Biology **74**, 91-103.

Pu CX, Ma Y, Wang J, Zhang YC, Jiao XW, Hu YH, Wang LL, Zhu ZG, Sun D, Sun Y. 2012. Crinkly4 receptor-like kinase is required to maintain the interlocking of the palea and lemma, and fertility in rice, by promoting epidermal cell differentiation. The Plant Journal **70**, 940-953.

Pu L, Li Q, Fan X, Yang W, Xue Y. 2008. The R2R3 MYB transcription factor GhMYB109 is required for cotton fiber development. Genetics **180**, 811-820.

Qi CH, Zhao XY, Jiang H, Zheng PF, Liu HT, Li YY, Hao YJ. 2019. Isolation and functional identification of an apple MdCER1 gene. Plant Cell, Tissue and Organ Culture (PCTOC) **136**, 1-13.

Qin BX, Tang D, Huang J, *et al.* 2011. Rice OsGL1-1 is involved in leaf cuticular wax and cuticle membrane. Molecular Plant **4**, 985-995.

Shan CM, Shangguan XX, Zhao B, *et al.* 2014. Control of cotton fibre elongation by a homeodomain transcription factor GhHOX3. Nature Communications **5**, 5519.

Shangguan X, Yang Q, Wu X, Cao J. 2021. Function analysis of a cotton R2R3 MYB transcription factor GhMYB3 in regulating plant trichome development. Plant Biology **23**, 1118-1127.

Shangguan XX, Yang CQ, Zhang XF, Wang LJ. 2016. Functional characterization of a basic helix-loop-helix (bHLH) transcription factor GhDEL65 from cotton (Gossypium hirsutum). Physiologia Plantarum **158**, 200-212.

Shi JX, Adato A, Alkan N, *et al.* 2013. The tomato SlSHINE3 transcription factor regulates fruit cuticle formation and epidermal patterning. New Phytologist **197**, 468-480.

Somaratne Y, Tian Y, Zhang H, Wang M, Huo Y, Cao F, Zhao L, Chen H. 2017. ABNORMAL POLLEN VACUOLATION1 (APV1) is required for male fertility by contributing to anther cuticle and pollen exine formation in maize. The Plant Journal **90**, 96-110.

Sun W, Gao D, Xiong Y, Tang X, Xiao X, Wang C, Yu S. 2017. Hairy leaf 6, an AP2/ERF transcription factor, interacts with OsWOX3B and regulates trichome formation in rice. Molecular Plant **10**, 1417-1433.

Taketa S, Amano S, Tsujino Y, *et al.* 2008. Barley grain with adhering hulls is controlled by an ERF family transcription factor gene regulating a lipid biosynthesis pathway. Proceedings of the National Academy of Sciences **105**, 4062-4067.

Vendemiatti E, Zsögön A, e Silva GFF, de Jesus FA, Cutri L, Figueiredo CRF, Tanaka FAO, Nogueira FTS, Peres LEP. 2017. Loss of type-IV glandular trichomes is a heterochronic trait in tomato and can be reverted by promoting juvenility. Plant Science **259**, 35-47.

Vendramin E, Pea G, Dondini L, *et al.* 2014. A unique mutation in a MYB gene cosegregates with the nectarine phenotype in peach. PLoS ONE **9**, e90574.

Walford SA, Wu Y, Llewellyn DJ, Dennis ES. 2011. GhMYB25-like: a key factor in early cotton fibre development. The Plant Journal **65**, 785-797.

Walford SA, Wu Y, Llewellyn DJ, Dennis ES. 2012. Epidermal cell differentiation in cotton mediated by the homeodomain leucine zipper gene, GhHD-1. The Plant Journal **71**, 464-478.

Wang H, Guo S, Qiao X, *et al.* 2019. BZU2/ZmMUTE controls symmetrical division of guard mother cell and specifies neighbor cell fate in maize. PLoS Genetics **15**, e1008377.

Wang S, Wang JW, Yu N, Li CH, Luo B, Gou JY, Wang LJ, Chen XY. 2004. Control of plant trichome development by a cotton fiber MYB gene. The Plant Cell **16**, 2323-2334.

Wang W, Liu X, Gai X, Ren J, Liu X, Cai Y, Wang Q, Ren H. 2015a. Cucumis sativus L. WAX2 plays a pivotal role in wax biosynthesis, influencing pollen fertility and plant biotic and abiotic stress responses. Plant and Cell Physiology **56**, 1339-1354.

Wang W, Zhang Y, Xu C, Ren J, Liu X, Black K, Gai X, Wang Q, Ren H. 2015b. Cucumber ECERIFERUM1 (CsCER1), which influences the cuticle properties and drought tolerance of cucumber, plays a key role in VLC alkanes biosynthesis. Plant Molecular Biology **87**, 219-233.

Wang X, Zhi P, Fan Q, Zhang M, Chang C. 2019. Wheat CHD3 protein TaCHR729 regulates the cuticular wax biosynthesis required for stimulating germination of Blumeria graminis f. sp. tritici. Journal of Experimental Botany **70**, 701-713.

Wang Y, Wan L, Zhang L, Zhang Z, Zhang H, Quan R, Zhou S, Huang R. 2012. An ethylene response factor OsWR1 responsive to drought stress transcriptionally activates wax synthesis related genes and increases wax production in rice. Plant Molecular Biology **78**, 275-288.

Wang Z, Guhling O, Yao R, Li F, Yeats TH, Rose JK, Jetter R. 2011. Two oxidosqualene cyclases responsible for biosynthesis of tomato fruit cuticular triterpenoids. Plant Physiology **155**, 540-552.

Wang Z, Tian X, Zhao Q, Liu Z, Li X, Ren Y, Tang J, Fang J, Xu Q, Bu Q. 2018. The E3 ligase DROUGHT HYPERSENSITIVE negatively regulates cuticular wax biosynthesis by promoting the degradation of transcription factor ROC4 in rice. The Plant Cell **30**, 228-244.

Weidenbach D, Jansen M, Franke RB, *et al.* 2014. Evolutionary conserved function of barley and Arabidopsis 3-KETOACYL-CoA SYNTHASES in providing wax signals for germination of powdery mildew fungi. Plant Physiology **166**, 1621-1633.

Wu H, Liu L, Chen Y, Liu T, Jiang Q, Wei Z, Li C, Wang Z. 2022. Tomato SlCER1–1 catalyzes the synthesis of wax alkanes, increasing drought tolerance and fruit storability. Horticulture Research **9**, uhac004.

Wu H, Tian Y, Wan Q, *et al.* 2018. Genetics and evolution of MIXTA genes regulating cotton lint fiber development. New Phytologist **217**, 883-895.

Wu R, Li S, He S, Waßmann F, Yu C, Qin G, Schreiber L, Qu LJ, Gu H. 2011. CFL1, a WW domain protein, regulates cuticle development by modulating the function of HDG1, a class IV homeodomain transcription factor, in rice and Arabidopsis. The Plant Cell **23**, 3392-3411.

Wu Z, Chen L, Yu Q, Zhou W, Gou X, Li J, Hou S. 2019. Multiple transcriptional factors control stomata development in rice. New Phytologist **223**, 220-232.

Xie K, Wu S, Li Z, *et al.* 2018. Map-based cloning and characterization of Zea mays male sterility33 (ZmMs33) gene, encoding a glycerol-3-phosphate acyltransferase. Theoretical and Applied Genetics **131**, 1363-1378.

Xie Q, Gao Y, Li J, Yang Q, Qu X, Li H, Zhang J, Wang T, Ye Z, Yang C. 2020. The HD-Zip IV transcription factor SlHDZIV8 controls multicellular trichome morphology by regulating the expression of Hairless-2. Journal of Experimental Botany **71**, 7132-7145.

Xie Y, Yu X, Jiang S, *et al.* 2020. OsGL6, a conserved AP2 domain protein, promotes leaf trichome initiation in rice. Biochemical and Biophysical Research Communications **522**, 448-455.

Xu J, van Herwijnen ZO, Dräger DB, Sui C, Haring MA, Schuurink RC. 2018. SlMYC1 regulates type VI glandular trichome formation and terpene biosynthesis in tomato glandular cells. The Plant Cell **30**, 2988-3005.

Xu Y, Liu S, Liu Y, Ling S, Chen C, Yao J. 2017. HOTHEAD-like HTH1 is involved in anther cutin biosynthesis and is required for pollen fertility in rice. Plant and Cell Physiology **58**, 1238-1248.

Xu Y, Wu H, Zhao M, Wu W, Xu Y, Gu D. 2016. Overexpression of the transcription factors GmSHN1 and GmSHN9 differentially regulates wax and cutin biosynthesis, alters cuticle properties, and changes leaf phenotypes in Arabidopsis. International Journal of Molecular Sciences **17**, 587.

Yang Q, Yang X, Wang L, *et al.* 2022. Two R2R3-MYB genes cooperatively control trichome development and cuticular wax biosynthesis in Prunus persica. New Phytologist **234**, 179-196.

Yang S, Cai Y, Liu X, *et al.* 2018. A CsMYB6-CsTRY module regulates fruit trichome initiation in cucumber. Journal of Experimental Botany **69**, 1887-1902.

Yang X, Zhang W, He H, *et al.* 2014. Tuberculate fruit gene Tu encodes a C2H2 zinc finger protein that is required for the warty fruit phenotype in cucumber (Cucumis sativus L.). The Plant Journal **78**, 1034-1046.

Yeats TH, Martin LB, Viart HM, *et al.* 2012. The identification of cutin synthase: formation of the plant polyester cutin. Nature Chemical Biology **8**, 609-611.

Yu D, Ranathunge K, Huang H, Pei Z, Franke R, Schreiber L, He C. 2008. Wax Crystal-Sparse Leaf1 encodes a β–ketoacyl CoA synthase involved in biosynthesis of cuticular waxes on rice leaf. Planta **228**, 675-685.

Yu Q, Chen L, Zhou W, An Y, Luo T, Wu Z, Wang Y, Xi Y, Yan L, Hou S. 2020. RSD1 is essential for stomatal patterning and files in rice. Frontiers in Plant Science **11**, 600021.

Zhai X, Wu H, Wang Y, *et al.* 2022. The fruit glossiness locus, dull fruit (D), encodes a C2H2-type zinc finger transcription factor, CsDULL, in cucumber (Cucumis sativus L.). Horticulture Research **9**, uhac146.

Zhang D, Yang H, Wang X, Qiu Y, Tian L, Qi X, Qu LQ. 2020. Cytochrome P450 family member CYP96B5 hydroxylates alkanes to primary alcohols and is involved in rice leaf cuticular wax synthesis. New Phytologist **225**, 2094-2107.

Zhang F, Zuo K, Zhang J, Liu X, Zhang L, Sun X, Tang K. 2010. An L1 box binding protein, GbML1, interacts with GbMYB25 to control cotton fibre development. Journal of Experimental Botany **61**, 3599-3613.

Zhang H, Wang Y, Tan J, Weng Y. 2022. Functional copy number variation of CsSHINE1 is associated with fruit skin netting intensity in cucumber, Cucumis sativus. Theoretical and Applied Genetics **135**, 2101-2119.

Zhang H, Wu K, Wang Y, Peng Y, Hu F, Wen L, Han B, Qian Q, Teng S. 2012. A WUSCHEL-like homeobox gene, OsWOX3B responses to NUDA/GL-1 locus in rice. Rice **5**, 1-10.

Zhang J, Yang J, Yang Y, Luo J, Zheng X, Wen C, Xu Y. 2019. Transcription factor CsWIN1 regulates pericarp wax biosynthesis in cucumber grafted on pumpkin. Frontiers in Plant Science **10**, 1564.

Zhang S, Wu S, Niu C, *et al.* 2021. ZmMs25 encoding a plastid-localized fatty acyl reductase is critical for anther and pollen development in maize. Journal of Experimental Botany **72**, 4298-4318.

Zhang YL, Zhang CL, Wang GL, Wang YX, Qi CH, You CX, Li YY, Hao YJ. 2019a. Apple AP2/EREBP transcription factor MdSHINE2 confers drought resistance by regulating wax biosynthesis. Planta **249**, 1627-1643.

Zhang YL, Zhang CL, Wang GL, Wang YX, Qi CH, Zhao Q, You CX, Li YY, Hao YJ. 2019b. The R2R3 MYB transcription factor MdMYB30 modulates plant resistance against pathogens by regulating cuticular wax biosynthesis. BMC Plant Biology **19**, 1-14.

Zhao G, Shi J, Liang W, Xue F, Luo Q, Zhu L, Qu G, Chen M, Schreiber L, Zhang D. 2015. Two ATP binding cassette G transporters, rice ATP binding cassette G26 and ATP binding cassette G15, collaboratively regulate rice male reproduction. Plant Physiology **169**, 2064-2079.

Zhao J, Long T, Wang Y, *et al.* 2020. RMS2 encoding a GDSL lipase mediates lipid homeostasis in anthers to determine rice male fertility. Plant Physiology **182**, 2047-2064.

Zhao L, Zhu H, Zhang K, Wang Y, Wu L, Chen C, Liu X, Yang S, Ren H, Yang L. 2020. The MIXTA-LIKE transcription factor CsMYB6 regulates fruit spine and tubercule formation in cucumber. Plant Science **300**, 110636.

Zheng F, Cui L, Li C, *et al.* 2022. Hair interacts with SlZFP8-like to regulate the initiation and elongation of trichomes by modulating SlZFP6 expression in tomato. Journal of Experimental Botany **73**, 228-244.

Zhou L, Ni E, Yang J, Zhou H, Liang H, Li J, Jiang D, Wang Z, Liu Z, Zhuang C. 2013. Rice OsGL1-6 is involved in leaf cuticular wax accumulation and drought resistance. PloS ONE **8**, e65139.

Zhou X, Jenks MA, Liu J, Liu A, Zhang X, Xiang J, Zou J, Peng Y, Chen X. 2014. Overexpression of transcription factor OsWR2 regulates wax and cutin biosynthesis in rice and enhances its tolerance to water deficit. Plant Molecular Biology Reporter **32**, 719-731.

Zhou X, Li L, Xiang J, Gao G, Xu F, Liu A, Zhang X, Peng Y, Chen X, Wan X. 2015. OsGL1-3 is involved in cuticular wax biosynthesis and tolerance to water deficit in rice. PLoS ONE **10**, e116676.

Zou LP, Sun XH, Zhang ZG, Liu P, Wu JX, Tian CJ, Qiu JL, Lu, TG. 2011. Leaf rolling controlled by the homeodomain leucine zipper class IV gene Roc5 in rice. Plant Physiology **156**, 1589-1602.
